# Associations between per- and polyfluoroalkyl substances (PFAS), DNA methylation and gene expression from background exposed Norwegian women (2003–2006)

**DOI:** 10.1038/s41598-026-45562-6

**Published:** 2026-04-11

**Authors:** Ana Carolina M. F. Coêlho, Torkjel M. Sandanger, Dorte Herzke, Charlotta Rylander, Vivian Berg, Therese Haugdahl Nøst

**Affiliations:** 1 Department of Community Medicine, Faculty of Health Sciences, UiT The Arctic University of Norway, Tromsø, Norway; 2 NILU, Tromsø, Norway; 3 Norwegian Institute for Public Health, Oslo, Norway; 4 Department of Laboratory Medicine, University Hospital of North Norway, Tromsø, Norway; 5 Department of Medical Biology, Faculty of Health Sciences, UiT The Arctic University of Norway, Tromsø, Norway; 6 Department of Public Health and Nursing, Norwegian University of Science and Technology, Trondheim, Norway

**Keywords:** PFAS, DNA methylation, Gene expression, Biomarkers, Genetics, Molecular biology, Risk factors

## Abstract

Per- and polyfluoroalkyl substances (PFAS) are a large group of widespread synthetic substances associated with several adverse health effects. A potential mechanism underlying these effects is epigenetic modifications, such as DNA methylation. However, evidence on associations between PFAS exposure and DNA methylation among adults is limited, and few studies have investigated the potential influence of PFAS-related methylation changes on gene expression. In this study, we investigated associations between PFAS exposure and DNA methylation in blood. Furthermore, the associations between these methylation differences and gene expression patterns were explored to assess potential functional effects. In addition, associations between PFAS concentrations and estimated white blood cell composition were investigated. This study included women (n = 269) from The Norwegian Women and Health Study (NOWAC), with available blood data on PFAS exposure, DNA methylation, and gene expression. We investigated epigenome-wide associations between PFAS exposure and DNA methylation at individual CpG sites using multivariable linear regression analyses. In a sensitivity analysis, the main models were additionally adjusted for smoking status. Furthermore, we assessed any associations between PFAS exposure and gene expression of genes mapped to CpG sites nominally associated with PFAS, also using multivariable linear regression. Finally, we examined associations between PFAS exposure and estimated white blood cell composition using multivariable linear regression models. Concentrations of PFUnDA were associated with DNA methylation at 21 unique CpG sites, comprising 17 positive and 4 negative associations. Sensitivity analyses did not substantially alter these findings. Furthermore, methylation levels at these sites did not follow a dose-dependent pattern, and the expression of genes mapped to these CpG sites was not significantly associated with exposure levels. No significant associations were observed between other PFAS (PFOA, PFNA, PFHxS, PFHpS, PFOS, and br-PFOS) and DNA methylation or gene expression. Associations between PFHxS, PFHpS, and PFOS with DNA methylation and gene expression were observed at the nominal significance level (*p* value < 0.001), but these associations were modest in magnitude, lacked evidence of dose-dependence, and did not remain significant after correction for multiple testing. No statistically significant associations were observed between PFAS exposure and estimated white blood cell composition. Our findings suggest that PFUnDA may influence DNA methylation at specific CpG sites in blood of Norwegian women, though these epigenetic patterns were not accompanied by corresponding patterns in gene expression. Overall, there was limited evidence linking other PFAS to DNA methylation or gene expression.

**Supplementary Information:**

The online version contains supplementary material available at 10.1038/s41598-026-45562-6.

## Introduction

Per- and polyfluoroalkyl substances (PFAS) constitute a large and diverse group of synthetic organic fluorine chemicals. These compounds have been extensively used for decades in a variety of consumer products and industrial applications due to their unique hydrophobic and hydrophilic properties^1^. In addition, PFAS are resistant to degradation due to the highly stable fluorine bond, which leads to their persistence in the environment^2,3^ and their tendency to bioaccumulate in various ecosystems and within the food chain. The large-scale production and global usage of PFAS have raised significant environmental and health concerns, as PFAS exposure has been associated with various adverse effects, including liver and testicular cancer, immune and endocrine and metabolic disrupting effects^4–6^. Emerging evidence suggests that PFAS exposure may influence immune function, including alterations in circulating immune cell profiles^7,8^, which may reflect immune modulation and systemic inflammation.

The mechanisms of action underlying these adverse health effects are still not fully understood. Epigenetic modifications have been suggested as a potential mechanism through which environmental pollutants may contribute to adverse health outcomes^9,10^. DNA methylation is the most extensively studied epigenetic regulatory mechanism of PFAS^10^. It involves the addition of a methyl group to a cytosine, predominantly at cytosine-guanine dinucleotide (CpG) sites, and plays a key role in regulating normal gene expression^5,9,11^. While most epidemiological studies have primarily focused on mother-infant cohorts to examine the impact of PFAS exposure of DNA methylation^9,10^, there is still a notable gap in studies investigating the associations between PFAS exposure and DNA methylation in adult populations. Some studies have consistently observed associations between PFAS exposure and DNA methylation at various CpG sites across diverse populations, biological matrices, and epigenetic platforms, although the specific CpG sites and direction of effects varied between studies^9,10,14–16^. For instance, associations between PFAS and DNA methylation were reported observed at multiple CpG sites in blood samples from women highly exposed to PFAS through drinking water in Ronneby (Sweden), with hypomethylation more common than hypermethylation in the high versus low exposure group comparison. Effect sizes were generally moderate^9^. Another study observed associations in adults with background plasma PFOA and PFOS concentrations, with generally modest effect sizes. For PFOA, both hypomethylation and hypermethylation were reported, whereas for PFOS, only positive associations were observed^10^. Nevertheless, few studies have investigated both epigenetic and gene expression alterations associations with PFAS.

In this study, we investigated associations between serum PFAS concentrations and epigenome-wide DNA methylation profiles among healthy women from The Norwegian Women and Health Study (NOWAC), sampled between 2003 and 2006 during the period of initial actions for the phase-out of certain legacy PFAS like PFOS. Furthermore, we also explored gene expression patterns associated with PFAS-related CpG sites to better understand potential functional consequences of these epigenetic changes. Lastly, we investigated associations between serum PFAS and estimated proportions of immune cell types in blood, both to explore alterations in circulating immune cell proportions and to assess whether immune cell composition may contribute to or help contextualize the observed changes in methylation patterns.

## Results

### Characteristics of the study population

Participants had a mean age of 54.7 years, with age ranging from 43 to 62 years old. The mean BMI was 25.6 kg/m^2^ (Table 1). Four PFAS (PFOA, PFHxS, lin-PFOS [hereafter referred as PFOS], and branched-PFOS [hereafter referred as br-PFOS]) were detected in all study participants. Additionally, PFHpS, PFNA and PFUnDA were detected in 98.1%, 98.9%, and 99.6% of the study participants (Table S1). The median concentrations of the PFAS were as follows: PFOS (11.5 ng/mL), br-PFOS (8.15 ng/mL), PFOA (2.93 ng/mL), PFHxS (1.04 ng/mL), PFNA (0.65 ng/mL), PFUnDA (0.37 ng/mL), and PFHpS (0.27 ng/mL) (Table S1). Spearman’s correlations were positive between several PFAS, with PFOS and br-PFOS showing the strongest positive correlation (p = 0.86), followed by PFHpS and br-PFOS (p = 0.83) (Figure S1).

**Table 1:** Characteristics of study participants (n = 269).

Variable^a^	Mean (± SD) or n (%)
Age, years	54.8 ± 4.3
BMI, kg/m^2^	25.6 ± 3.9
Parity	
Nulliparous	21(7.8)
Primiparous	25 (9.3)
Multiparous	223 (82.9)
Duration of breastfeeding, months	14.1 ± 12.3
Smoking status	
Never smoker	101 (37.5)
Former smoker	82 (30.5)
Current smoker	80 (29.8)

^a^Values are means (SD) or n (percentages).

### Associations between PFAS concentration and epigenome-wide methylation

**Fig. 1:**
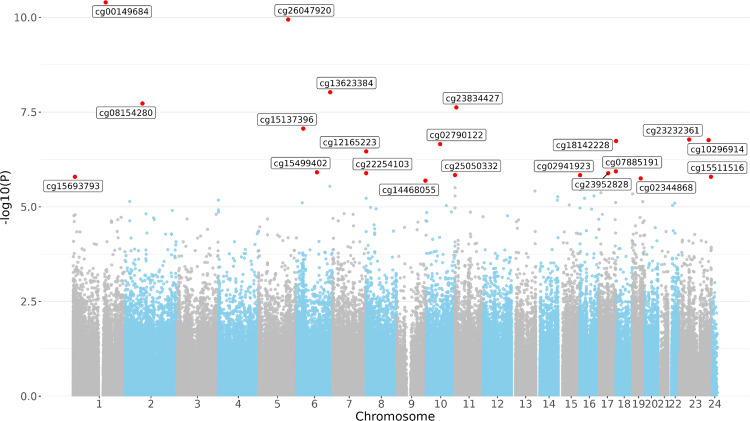
Epigenome-wide analysis of CpG methylation associated with PFUnDA exposure. Manhattan plot displaying the distribution of − log_10_ (*P*-value) across the genome for all CpG sites analyzed. Significant CpG sites are highlighted and shown in red.

**Fig. 2:**
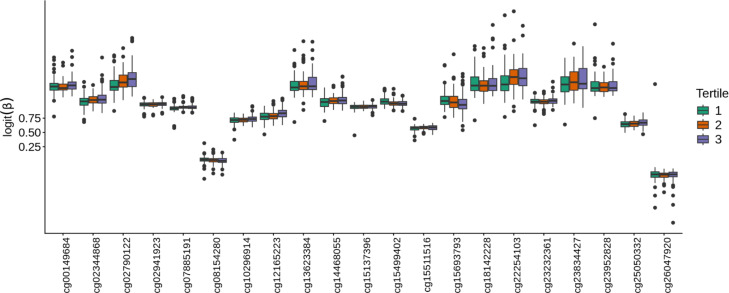
Epigenome-wide CpG methylation differences across PFUnDA exposure tertiles. Boxplots showing methylation levels of the significant CpG sites across tertiles of PFUnDA concentrations. Sorted according to increasing p-values from left to right.

**Fig. 3:**
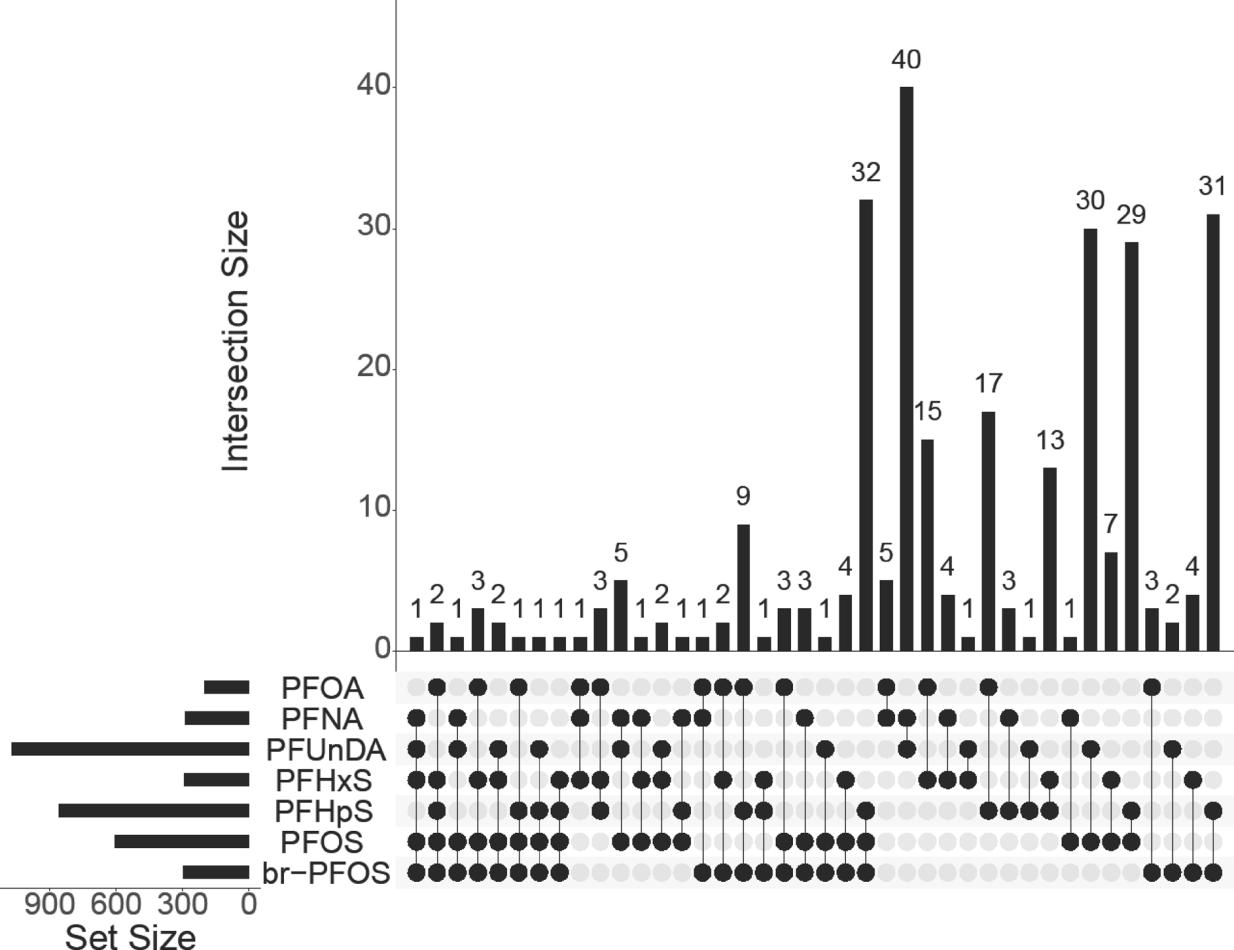
UpSet plot showing the overlap in the number of CpG sites (*p* value < 0.001) associated with different PFAS compounds. The bar chart illustrates the number of CpG sites (*p* value < 0.001) associated with various combinations of PFAS. Below, the graphical table identifies the specific PFAS combinations through black dots and connecting lines. On the left, a smaller bar chart displays the total frequency of each PFAS across the combinations presented in the plot.

A total of 21 CpG sites were observed to be significantly associated with plasma concentrations of PFUnDA in the epigenome-wide analysis, each with FDR < 0.05 (Table 2). Specifically, PFUnDA concentrations were negatively associated with methylation at four CpG sites and positively associated with methylation at 17 CpG sites (Figures S2). Fourteen of the CpG sites had beta values > 0.75, and the 21 CpG sites were located at 13 different chromosomes (Fig. 1). The correlations within the 21 significant CpG sites indicated weak to moderate, but significant, positive correlations between eight of these sites. These correlations ranged from 0.14 to 0.55, with the strongest correlation observed between *cg07885191* and *cg14468055* (Figure S3). When evaluating whether there were any differences in methylation levels of these 21 CpG sites across the PFUnDA concentration tertiles, no significant differences were observed between the highest (median = 0.60 ng/mL) and lowest (median = 0.22 ng/mL) tertiles (Fig. 2; Figure S4). There were no significant associations between other PFAS (PFOA, PFNA, PFHxS, PFHpS, PFOS and br-PFOS) and DNA methylation at individual CpG sites, regardless of whether PFAS concentrations were analyzed as a continuous variable or as tertiles (Figures S5-S10). Furthermore, when examining CpG sites that were nominally significant (i.e., non-FDR-corrected p-value < 0.001) (n = 3612), we observed overlapping associations between individual PFAS and DNA methylation at 282 specific CpG sites. The largest overlap was observed for PFNA and PFUnDA, with both being associated with the same 40 CpG sites. However, none of these specific CpG sites were consistently associated with all PFAS (Fig. 3). The model estimates for these epigenome-wide associations are provided in Tables S2-S7.

**Table 2:** CpG sites showing methylation changes associated with PFUnDA concentrations^*^.

CpG	Chr	Position	CpG location	Genes	Gene region	logFC	p-value	q-value
cg00149684	1	155,097,375	N_Shore	-	-	0.01	0.00000000004	0.00002
cg26047920	5	141,303,242	Island	*DELE1*	TSS200^a^	-0.04	0.0000000001	0.00003
cg13623384	6	160,176,839	OpenSea	*WTAP*	3’UTR^b^	0.02	0.000000009	0.001
cg08154280	2	80,530,948	N_Shore	*CTNNA2; LRRTM1*	Body; 5’UTR^c^	-0.01	0.00000002	0.002
cg23834427	11	6,542,019	OpenSea	*DNHD1*	3’UTR^b^; Body	0.02	0.00000002	0.002
cg15137396	6	32,039,841	OpenSea	*TNXB*	Body	0.02	0.00000009	0.007
cg23232361	X	48,619,052	OpenSea	*GLOD5*	TSS1500^d^	0.01	0.0000002	0.01
cg10296914	X	140,980,959	OpenSea	*MAGEC3*	Body; TSS1500^d^	0.03	0.0000002	0.01
cg18142228	18	581,171	S_Shore	*CETN1*	3’UTR^b^;1stExon	0.02	0.0000002	0.01
cg02790122	10	65,133,532	OpenSea	*JMJD1C; MIR1296*	Body; TSS1500^d^	0.01	0.0000002	0.01
cg12165223	8	980,196	S_Shore	*-*	-	0.03	0.0000003	0.01
cg07885191	17	80,848,209	OpenSea	*TBCD*	Body	0.02	0.000001	0.04
cg15499402	6	97,282,253	N_Shelf	*GPR63*	5’UTR^c^	-0.01	0.000001	0.04
cg23952828	17	44,246,087	OpenSea	*KANSL1*	Body	0.01	0.000001	0.04
cg22254103	8	989,193	Island	*-*	-	0.02	0.000001	0.04
cg25050332	11	316,088	Island	*-*	-	0.03	0.000001	0.04
cg02941923	16	1,005,077	N_Shore	*LMF1*	Body	0.01	0.000001	0.04
cg15511516	X	151,999,486	Island	*NSDHL;CETN2*	TSS200^a^;TSS200^a^	0.02	0.000002	0.04
cg15693793	1	8,880,657	S_Shelf	*-*	-	-0.03	0.000002	0.04
cg02344868	19	39,691,009	Island	*NCCRP1*	Body	0.02	0.000002	0.04
cg14468055	9	136,003,437	OpenSea	*RALGDS*	Body	0.01	0.000002	0.04

Chr, chromosome; FC, fold change; q-value, False discovery rate (FDR)-adjusted p-value using the Benjamini–Hochberg method.

^a^ Transcription start site 200 bp upstream of the gene [TSS200].

^b^ 3′ Untranslated regions (3’ UTR).

^c^ 5′ Untranslated regions (5’ UTR).

^d^ Transcription start site 1500 bp upstream of the gene [TSS1500].

The CpG sites were ranked in ascending order based on their unadjusted p-value (*).

### Sensitivity analysis

After adjusting for smoking status, the top 10 of the 21 CpG sites remained statistically significant with consistent direction and magnitude of effect (Table S8). The remaining 11 CpG sites did not reach the statistical significance (FDR > 0.05), although the direction and magnitude of effect remained mostly unchanged. Specifically, seven of these sites had an FDR ≤ 0.10, and three had slightly higher FDR values. These findings indicate that the main findings are robust to the inclusion of smoking status, although some associations become less statistically significant when this additional covariate was considered.

### Functional enrichment analysis

A global evaluation of all nominally significant (i.e., non-FDR-corrected p-value < 0.001) CpG sites associated with PFAS exposure (n = 3,612) resulted in 193 biological process (BP), 42 molecular function (MF), and 36 cellular component (CC) terms. The top five GO terms of BP included heart development (GO:2,000,136, GO:2,000,138, and GO:0,061,323), cellular processes (GO:2,001,222), and histone modification (GO:2,000,617). However, none of these GO terms were statistically significant for any PFAS after adjusting for multiple comparisons. All top enriched GO-terms for each of three categories (BP, MF, and CC) are provided in Table S9.

### Associations between PFAS concentrations and gene expression

After FDR correction, no significant associations were found between PFUnDA concentrations and the expression of the genes mapped from the 21 CpG sites with significant DNA methylation changes associated with PFUnDA. Of the 16 CpGs annotated to genes (19 genes total), expression data were available for 8 genes. Additionally, we examined correlations between DNA methylation and gene expression for the annotated CpG–gene pairs. Spearman correlation analyses showed no strong associations between DNA methylation and gene expression (Figure S11). To further investigate, we expanded the analysis to include the genes mapped to nominally significant (i.e., non-FDR-corrected p-value < 0.001) CpG sites associated with PFAS exposure (n = 3,612). After FDR correction, no significant associations were observed. However, among the nominally significant (i.e., non-FDR-corrected p-value < 0.01) results, we observed that PFHpS concentrations were associated with seven genes (*NCOA4*, *SMYD3*, *CSE1L*, *ZXDC*, *PTPRJ*, *RHOC*, and *STAT5B*), PFHxS concentrations associated with two genes (*C1orf124* and *TFDP1*), and PFOS concentrations associated with four genes (*AATK*, *RND2*, *GPR146*, and *ZFYVE2*) (Table S10). No significant associations were observed between the concentrations of PFAS and genome-wide gene expression.

### Associations between PFAS concentrations and estimated white blood cell composition

Plasma concentrations of any PFAS, after adjusting for age and BMI, were not significantly associated with the estimated white blood cell proportions, except for PFOA, showing a weak positive association with B lymphocytes proportions (p-value < 0.05) (Table S11).

## Discussion

In this study, we observed that PFUnDA was the only PFAS significantly associated with DNA methylation for 21 unique CpG sites in blood of Norwegian women. Of these, 17 CpG sites were positively associated with PFUnDA concentrations, while 4 CpG sites were negatively associated. Nevertheless, the magnitude of these effects was small. Moderate correlations were observed between eight of the significant CpG sites, suggesting that these sites may be regulated by common biological mechanisms. However, the correlations were not strong enough to indicate a clear, unified pattern of methylation differences across these sites. The 21 significant CpG sites identified in this study were distributed across multiple chromosomes and annotated to diverse genomic regions, including promoter-associated regions (transcription start site 200 bp upstream of the gene [TSS200] and 1500 bp upstream [TSS1500]), 5′ and 3′ untranslated regions (UTRs), the first exon, and gene body regions, suggesting potential regulatory roles in gene expression. However, we did not observe any significant differences in the expression of these genes in relation to PFUnDA concentrations. Additionally, no significant associations were found between any other PFAS (PFOA, PFNA, PFHxS, PFHpS, PFOS, and br-PFOS) and DNA methylation at individual CpG sites, nor between PFAS exposure and genome-wide gene expression. Furthermore, PFAS exposure was not associated with estimated white blood cell proportions, suggesting that PFAS does not appear to influence white blood cell composition in this cohort.

The associations between PFUnDA and DNA methylation at 21 CpG sites observed in this study are novel. Although few studies have specifically examined PFUnDA, previous research has reported associations between long-chain PFAS (PFNA, PFDA) and DNA methylation, suggesting that these compounds may influence DNA methylation even at relatively low exposure levels^15,16^. The limited number of PFUnDA-focused studies, particularly in populations with background exposure^17^, which limits direct comparisons. A study conducted on firefighters (n = 197), who can be highly exposed to PFAS from occupational practices, identified one CpG site associated with PFUnDA (*cg01721356*, annotated to *LOC339529*)^13^, while a study conducted in pregnant women (n = 260) observed strong associations between higher concentrations of PFUnDA, DNA methylation (*cg13996963*, *cg12089439*, and *cg18145877*, annotated to tumor suppressor candidate 3 [*TUSC3]*), and gene expression (*TUSC3*)^18^. However, we did not observe associations with DNA methylation at the same sites in the present study, nor with gene expression related to TUSC3. In contrast, our findings suggest that while DNA methylation changes may occur in response to PFUnDA exposure, these changes may not directly correlate with gene expression within the same samples. Additional regulatory factors, such as non-CpG methylation and histone acetylation, could also influence gene expression, emphasizing the complexity of transcriptional regulation beyond DNA methylation alone^19^. Although PFUnDA concentrations were lower than those of more prevalent PFAS like PFOS in this study population, its distinct chemical structure and emerging evidence of health effects^20,21^ suggest it could still have important biological implications. Notably, time trends have indicated increasing concentrations of PFUnDA in populations from western countries since the sampling for this study was conducted^22–24^, highlighting the need for further research on its potential health impacts, particularly in relation to blood lipids, metabolism, and developmental outcomes^25–27^.

When considering nominally significant (i.e., non-FDR-corrected p-value < 0.001) CpG sites associated with PFAS exposure (n = 3,612), we observed that multiple PFAS were associated with several of the same CpG sites among these. For instance, we observed that PFNA, PFUnDA, PFHxS, PFOS, and br-PFOS were associated with *cg07601058*, annotated to solute carrier family 35 member E2B *(SLC35E2B*; gene body), a member of the solute carrier family involved in transmembrane transport, and cyclin dependent kinase 11B *(CDK11B;* gene body, 5’UTR), which plays a role in cell cycle regulation and transcriptional control. PFOA, PFHxS, PFHpS, PFOS, and br-PFOS were associated with two additional CpG sites: *cg19882132*, which is currently not annotated to a known gene, and *cg23067082*, annotated to microRNA 141 *(MIR141*, TSS200), involved in post-transcriptional regulation of gene expression in multicellular organisms by affecting mRNA stability and translation. The magnitude and direction of the associations among these CpG sites were consistent across the PFAS but did not overlap with observations in other studies. Overlapping associations between multiple PFAS and the same CpG sites may indicate a shared underlying mechanism or pathway, warranting further investigation to identify the specific biological processes involved and to understand how these PFAS exposures might jointly influence these sites to influence gene expression or other molecular functions.

We further mapped the nominally significant CpG sites (i.e., non-FDR-corrected p-value < 0.001) associated with each PFAS to their corresponding genes. Blood gene expression levels within the same samples were then examined to explore potential PFAS-related transcriptional influences. Although the associations between PFAS exposure and gene expression did not reach statistical significance after FDR correction, 13 genes were nominally significant (p-value < 0.01) prior to correction, with associations observed for three PFAS: PFHxS, PFHpS, and PFOS. However, none of these 13 genes have been reported in other human studies investigating PFAS-associated epigenetic or transcriptomic changes. Still, certain genes may be of interest not merely due to the modest associations between PFAS concentrations, DNA methylation, and their expression, but because of their biological function. As an example, one of these genes was the G protein-coupled receptor 146 (*GPR146)*, which was negatively associated with PFOS in our study. This gene has been linked to the regulation of blood cholesterol levels in humans, and its downregulation may result in decreased blood cholesterol levels^29,30^. Previous studies have reported disruption of lipid metabolism related to PFAS exposures^26,27^. In a Norwegian population (the Tromsø Study), PFAS exposures were associated with total cholesterol levels over time, further supporting a relationship between PFAS and lipid metabolism disruptions^25^. Our findings could suggest that PFAS-related health effects could involve small and complex interactions between epigenetic modifications and transcriptional changes, highlighting the need for further research to understand these mechanisms and their potential implications for health outcomes.

Studies investigating the relationship between PFAS exposure and DNA methylation in adults are limited, and most have focused on populations with higher PFAS exposure levels than those observed in the Norwegian women in this study. For example, a study conducted in a highly exposed group of women (n = 64) from Ronneby, Sweden, identified 117 differentially methylated CpG sites associated with PFAS (PFHxS, PFOA, and PFOS), with hypomethylation being more common than hypermethylation^9^. However, PFAS concentrations (especially PFOS and PFHxS) in the Ronneby cohort were considerably higher than those observed in the present study (e.g. the median PFOS concentration in the Ronneby cohort was 230 ng/mL, compared to 11.5 ng/mL in our study). Furthermore, the study conducted among firefighters demonstrated that branched-PFOA, linear-PFOS, PFNA, PFDA, and PFUnDA were associated with differentially methylated loci and regions^13^. However, the previously reported CpG sites and the magnitude of associations did not overlap with those observed in the present study. The closest observation was the identification of a CpG site annotated to the tenascin-XB gene (*TNXB*) associated with PFOA and PFOS in newborns from US The Upstate KIDS study^31^. Still, the specific CpG site within the *TNXB* gene differed and both directions of effects were observed. The inconsistencies between findings may be explained by multiple factors, including study population and design, sample size/statistical power, covariates adjusted, data analysis methods, concentration ranges and timing of PFAS exposure measurements, and the timing and methods of measuring DNA methylation^17,32^. PFAS concentrations in the Norwegian women in our study were generally consistent with background exposure levels in other background-exposed, population-based adult cohorts sampled during the same time period, such as NHANES in the US^33,34^, the Tromsø Study in Norway^35^, and the Danish National Birth Cohort^36^.

There are important strengths in the present study that should be noted. First, both DNA methylation and gene expression data were available for a large sample of participants, with both data types obtained from the same individuals, and all data collected at the same time point. Additionally, our study is based on samples collected during a period when PFAS concentrations were near peak levels for certain compounds in this background-exposed population, representing a time of potentially highest exposure. Moreover, the study also included a range of several PFAS, including those present at relatively low concentrations.

The present study has several notable limitations. First, the study design is cross-sectional, meaning that PFAS concentrations, DNA methylation, and gene expression were measured at a single time point. This design may limit the ability to capture temporal variations, especially in gene expression, which can fluctuate dynamically. Second, our significant findings were primarily for PFUnDA, which is present at relatively low concentrations in this population. This may have limited the statistical power of the study, and we cannot rule out the possibility that some findings occurred by chance. However, multiple testing corrections were applied to reduce the risk of false positives. Third, the study population was limited to Norwegian women within a specific age range who were exposed to background concentrations of PFAS, which may affect the generalizability of the findings. Moreover, including only women who remained cancer-free throughout follow-up may have influenced the results, as PFAS-related epigenetic effects could differ in women who later developed cancer. Fourth, our analyses focused on individual PFAS, which may not fully capture the combined or interactive effects of co-occurring PFAS. Notably, the single-compound analyses revealed only limited associations, indicating that mixture effects may be modest or require larger sample sizes to detect. Additionally, the mechanisms of action of PFAS are not fully understood and may differ across compounds due to variations in chemical properties such as chain length, functional groups, and bioaccumulation potential. Finally, the DNA methylation data analyzed in this study were obtained using the Illumina 450 K array, which measures the average methylation level across all CpG sites but cannot distinguish between 5-methylcytosine and 5-hydroxymethylcytosine, previously associated with PFAS exposures^15^. While this platform was appropriate at the time of data generation, more comprehensive arrays (e.g., EPIC 850 K, EPIC v2 ~ 930 K) are now standard, and our study may have limited CpG coverage compared with these newer platforms.

In this study, concentrations of PFUnDA were associated with DNA methylation at 21 unique CpG sites in Norwegian women. Still, no strong associations nor specific genomic region were indicated and no accompanying significant changes in gene expression were observed. This investigation targeted associations between PFAS, DNA methylation and gene expression in a background-exposed population during a period of declining exposure to certain legacy PFAS.

## Material and methods

### Study cohort

The study participants were selected from the NOWAC postgenome cohort, which represents a subset of the nation-wide population-based NOWAC cohort [detailed information have been previously described in^37^]. The NOWAC postgenome cohort consists of approximately 50,000 women, who were invited to donate blood samples between 2003 and 2006^30^. All participants have answered questionnaires regarding medical information and lifestyle.

**Fig. 4:**
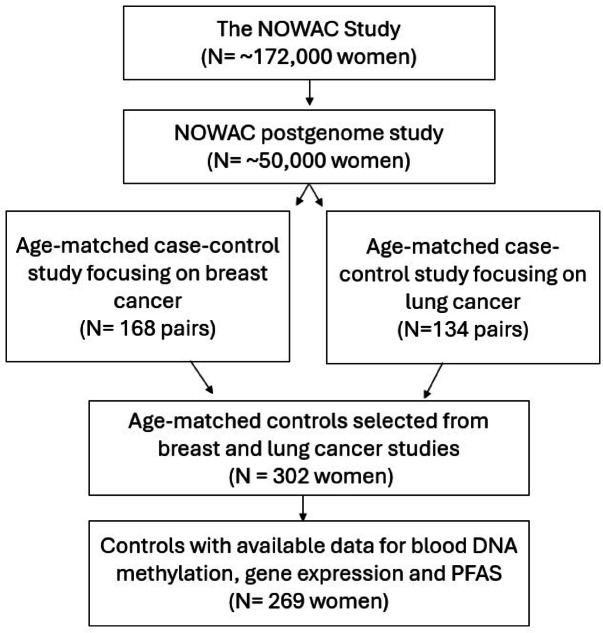
Flowchart of sampling procedure and exclusion criteria. This figure shows how the final sample for this study was selected. Study participants were selected from the NOWAC postgenome cohort, a subset of the nation-wide population-based NOWAC cohort. The postgenome cohort includes approximately 50,000 women who donated blood samples between 2003 and 2006. The present study included 302 women who previously served as age-matched controls in earlier case–control studies and had DNA methylation and gene expression data available. Women with missing DNA methylation or gene expression data were excluded, resulting in a final analytic sample of 269 cancer-free women with both DNA methylation and gene expression measurements.

The present study included a subset of 302 women who previously served as age-matched controls in earlier case–control studies^39,40^, with DNA methylation and gene expression data available. These participants were cancer-free at the time of enrollment, during blood sample collection (2003–2006), and remained cancer-free until the end of the follow-up period, which varied depending on the specific study. Women lacking complete anthropometric information (age, weight, or height) or with missing DNA methylation data or gene expression were excluded, resulting in a final study sample of 269 participants with both DNA methylation and gene expression measurements (Fig. 4).

Participation in the NOWAC Study was voluntarily, and a signed consent form was obtained from all participants. The present study was approved by the Regional Committee for Medical Research Ethics (REK, case number: 2020/154,446).

### PFAS analysis

Targeted PFAS analysis was conducted on plasma samples as previously described in^41^. Briefly, the analysis quantified 16 legacy perfluoroalkyl acids (PFAA), including seven PFSA (C4-C10) and nine PFCA (C4, C7-C13), as well as four known PFAA precursors (perfluorooctane sulfonamide [FOSA] and three fluorotelomers sulfonic acids [FTSA]: 4:2 FTS, 6:2 FTS, and 8:2 FTS). Samples were processed with isotopically labeled internal standards, extracted with methanol, and analyzed using ultra-high pressure liquid chromatography coupled with triple-quadrupole mass spectrometry (UHPLC-MS/MS). A detailed description of the PFAS analysis, including information regarding chemicals, standards, and quality control procedures, is provided elsewhere^42^, including information regarding chemicals, standards, and quality control procedures.

### DNA methylation and gene expression microarray data

Genome-wide DNA methylation analyses have previously been described in detail elsewhere^40,43,44^. Briefly, genomic DNA was extracted from buffy coats and hybridized to Illumina Infinium HumanMethylation450 BeadChip according to manufacturer’s protocol. The raw data were processed using an in-house code written in the R statistical software, which included data filtering, background subtraction, and dye bias correction^43^. Following quality control, a total of 482,421 CpG sites were retained and included in the final modeling analyses*.*

For the same study participants, total RNA was extracted and purified from PAXgene Blood RNA tubes using the PAXgene Blood RNA kit protocol. RNA integrity and purity were assessed, and complementary RNA (cRNA) was synthesized and hybridized to Illumina WG-3 or HT-12 expression bead chips for gene expression profiling. The raw expression data were processed in Illumina GenomeStudio, with background correction, log2 transformation, and quantile normalization applied. Detailed information on the analytical procedures and preprocessing steps is provided elsewhere^45^. Following quality control, for genes represented by multiple probes, the probe with the highest interquartile range (IQR) was retained, resulting in 7,247 unique gene-level expression values included in the final analyses.

### Covariates

The models constructed to evaluate the associations between PFAS and DNA methylation and gene expression included covariates selected based on prior knowledge: age, body mass index (BMI), and estimated proportions of specific white blood cells. Previous studies have shown that these factors are associated with both PFAS exposure and epigenetic or gene expression variation, making them important potential confounders to consider^7–9,15,46^. The proportions of monocytes, CD4 T-cells, CD8 T-cells, natural killers (NK) cells, B lymphocytes, neutrophils and eosinophils were previously estimated from the DNA methylation data^40,44^ using Houseman’s reference-based deconvolution algorithm^46^.

### Statistical analysis

All data processing and statistical analyses were conducted using R version 4.1.2 (R Core Team). Plasma concentrations of PFAS were log-transformed prior to the analysis. For plasma concentrations of PFAS below the LOD, the values were substituted by LOD/√2. The statistical analyses included seven PFAS compounds (PFOA, PFNA, PFUnDA, PFHxS, PFHpS, linear-PFOS, and branched-PFOS), all of which had a detection frequency above 90%. Spearman’s rank correlation analysis was performed to assess the relationship between the PFAS concentrations.

We first conducted an epigenome-wide analysis to evaluate associations between PFAS exposure and any DNA methylation levels by fitting a multivariable linear regression model to each CpG site using the *limma* package^47^, adjusting for the previously described covariates. To assess the impact of smoking status, a sensitivity analysis was performed by including it as an additional covariate for the significant CpG sites identified, to determine whether its inclusion influenced the estimates from the main model. In addition, we further investigated the impact of PFAS exposure on DNA methylation by categorizing plasma PFAS concentrations into tertiles. This categorization allowed us to evaluate differentially methylated CpG sites by comparing methylation levels across these exposure groups. The lowest tertile served as the reference group for conducting pairwise comparisons with the other tertiles, using a contrast matrix to identify significant differences. Unadjusted p-values from these comparisons were adjusted for multiple testing using the Benjamini–Hochberg false discovery rate (FDR) method, with a threshold of FDR < 0.05 considered statistically significant.

In a subsequent analysis, we focused on nominally significant CpG sites (i.e., non-FDR-corrected p-value < 0.001) to ensure a sufficient number of CpG sites for downstream analyses. These CpG sites were mapped to their respective gene names using the IlluminaHumanMethylation450kanno.ilmn12.hg19 annotation in the *minfi* package^48^. These annotated genes were also submitted to functional enrichment analysis for Gene Ontology (GO) terms, using *gometh* function from the *missMethyl* package^49^. Further, gene expression levels for these annotated, targeted genes were then investigated in relation to individual PFAS, using samples collected from the same individuals at the same time to ensure alignment of methylation and expression data. These statistical analyses were also performed using the *limma* package^47^, and p-values were corrected for multiple testing using the FDR method. Additionally, genome-wide association analyses were also performed to explore the relationship between individual PFAS and any gene expression levels, providing a comprehensive evaluation of PFAS-related epigenetic and transcriptomic associations.

DNA methylation patterns in blood samples are strongly influenced by cell type composition, which itself may be affected by PFAS exposure. We examined any associations between estimated white blood cell composition and PFAS concentrations using multivariable linear regression models for each cell type and each PFAS, adjusting for the covariates described above.

## Supplementary Information

Below is the link to the electronic supplementary material.

Supplementary Material 1

## Data Availability

Data from the NOWAC Study is available for research purposes through an application to the NOWAC access committee. Due to legal, privacy and ethical restrictions, the data cannot be made publicly available in open repositories. Detailed descriptions of variables and their metadata, including descriptions of data collection methods, variable definitions, are accessible at [https://helsedata.no] (https:/eur01.safelinks.protection.outlook.com/?url=https%3A%2F%2Fhelsedata.no%2F&data=05%7C02%7Ctonje.braaten%40uit.no%7C90a155431b0245a96ff008dda4258e43%7C4e7f212d74db4563a57b8ae44ed05526%7C0%7C0%7C638847203392359066%7CUnknown%7CTWFpbGZsb3d8eyJFbXB0eU1hcGkiOnRydWUsIlYiOiIwLjAuMDAwMCIsIlAiOiJXaW4zMiIsIkFOIjoiTWFpbCIsIldUIjoyfQ%3D%3D%7C0%7C%7C%7C&sdata=9F6aKXgYlDtIfJ%2F1%2Bu0qKnJKMPy0MrS5SKKXQJYwNJU%3D&reserved=0).

## Code availability

The source code is publicly available at https://github.com/pfas-omics/pfas-dnam-genex-wbc.
