# Supplementary material for: Associations between per- and polyfluoroalkyl substances (PFAS), DNA methylation and gene expression from background exposed Norwegian women (2003–2006)
Source: Sci Rep. 2026 Apr 11;16:17095. doi: 10.1038/s41598-026-45562-6 (PMC13230595; doi:10.1038/s41598-026-45562-6)
Supplement: Supplementary file 1 — Supplementary Material 1 [file 41598_2026_45562_MOESM1_ESM.docx]

**Associations between per- and polyfluoroalkyl substances (PFAS), DNA methylation and gene expression from background exposed Norwegian women (2003-2006)**

Ana Carolina M F Coêlho^1^, Torkjel M Sandanger^1,2^, Dorte Herzke^2,3^, Charlotta Rylander^1^, Vivian Berg^4,5^, Therese Haugdahl Nøst^1,6^

^1^ Department of Community Medicine, Faculty of Health Sciences, UiT The Arctic University of Norway, Tromsø, Norway

^2^ NILU, Tromsø, Norway

^3^ Norwegian Institute for Public Health, Oslo, Norway

^4^ Department of Laboratory Medicine, University Hospital of North Norway, Tromsø, Norway

^5^ Department of Medical Biology, Faculty of Health Sciences, UiT The Arctic University of Norway, Tromsø, Norway

^6^ Department of Public Health and Nursing, Norwegian University of Science and Technology, Trondheim, Norway

* Corresponding author: therese.h.nost@uit.no

**Table S1. Plasma PFAS concentrations (ng/mL) among study participants (n=269).**

| **PFAS** | **Median [P5, P95]** | **LOD** | **%DR** |
| --- | --- | --- | --- |
| **PFCA** | | | |
| PFBA | <LOD [<LOD, <LOD] | 0.47 | 1.9 |
| PFHpA | <LOD [<LOD, 0.14] | 0.06 | 37.9 |
| PFOA | 2.93 [1.40, 5.45] | 0.13 | 100 |
| PFNA | 0.65 [0.35, 1.15] | 0.24 | 98.9 |
| PFDA | 0.21 [<LOD, 0.67] | 0.02 | 52.4 |
| PFUnDA | 0.37 [0.13, 0.83] | 0.02 | 99.6 |
| PFDoDA | 0.04 [0.13, 0.13] | 0.02 | 60.6 |
| PFTrDA | <LOD [<LOD ¸0.17] | 0.07 | 31.2 |
| **PFSA** | | | |
| PFBS | <LOD [<LOD, 0.1] | 0.04 | 28.2 |
| PFPS | <LOD [<LOD, <LOD] | 0.07 | 1.9 |
| PFHxS | 1.04 [0.40, 2.25] | 0.11 | 100 |
| PFHpS | 0.27 [0.07, 0.65] | 0.03 | 98.1 |
| PFOS | 11.49 [5.21, 24] | 0.02 | 100 |
| br-PFOS | 8.15 [3.32, 16.27] | 0.26 | 100 |
| PFNS | <LOD [<LOD, 0.46] | 0.04 | 17.1 |
| PFDS | <LOD [<LOD, 0.12] | 0.05 | 16 |
| **FOSA** | | | |
| PFOSA | <LOD [<LOD, <LOD] | 0.06 | 0.37 |
| **FTSA** | | | |
| 4:2 FTS | <LOD [<LOD, <LOD] | 0.06 | 0 |
| 6:2 FTS | <LOD [<LOD, <LOD] | 0.06 | 0.37 |
| 8:2 FTS | <LOD [<LOD, 0.1] | 0.06 | 5.6 |

The grey shading indicates the PFAS compounds included in statistical analyses. Abbreviations: P: percentile; LOD: limit of detection; DR: detection rate; PFCA: perfluoroalkyl carboxylic acids; PFSA: perfluoroalkyl sulfonic acids; PFOSA: perfluorooctane sulfonamide; FTSA: fluorotelomer sulfonic acids.

**Table S2. Top 20 CpG sites showing methylation changes associated with PFOA concentrations*.**

| **CpG** | **Chr** | **Position** | **CpG location** | **Genes** | **Gene region** | **logFC** | **p-value** | **q-value** |
| --- | --- | --- | --- | --- | --- | --- | --- | --- |
| cg10724632 | 2 | 3675155 | OpenSea | *COLEC11* | Body | -0.16 | 0.00001 | 0.99 |
| cg03108697 | 11 | 9732066 | OpenSea | *SWAP70* | Body | 0.02 | 0.00002 | 0.99 |
| cg03134285 | 1 | 67836673 | OpenSea | *IL12RB2* | Body | 0.01 | 0.00003 | 0.99 |
| cg16359034 | 16 | 2795664 | OpenSea | *LOC100128788* | Body | 0.02 | 0.00003 | 0.99 |
| cg01458752 | 11 | 8008673 | N_Shore | *EIF3F* | TSS200 | -0.01 | 0.00003 | 0.99 |
| cg04011925 | 8 | 106671637 | OpenSea | *ZFPM2* | Body | 0.02 | 0.00003 | 0.99 |
| cg10367244 | 15 | 96865032 | Island | *-* | - | 0.01 | 0.00004 | 0.99 |
| cg21879272 | 14 | 51135303 | Island | *SAV1* | TSS1500 | -0.06 | 0.00004 | 0.99 |
| cg14442557 | 11 | 82909565 | S_Shelf | *ANKRD42* | Body | 0.01 | 0.00005 | 0.99 |
| cg23173910 | 2 | 74120274 | OpenSea | *ACTG2* | 5'UTR | 0.02 | 0.00005 | 0.99 |
| cg00923678 | 6 | 29581105 | OpenSea | *GABBR1* | Body; | 0.01 | 0.00005 | 0.99 |
| cg10857398 | 6 | 31598445 | N_Shore | *BAT2* | Body | 0.01 | 0.00005 | 0.99 |
| cg11815438 | 6 | 32023283 | OpenSea | *TNXB* | Body | 0.01 | 0.00006 | 0.99 |
| cg17476271 | 11 | 128562408 | N_Shore | *FLI1* | TSS1500 | -0.02 | 0.00006 | 0.99 |
| cg23434654 | 5 | 121648409 | Island | *SNCAIP* | 5'UTR | -0.01 | 0.00006 | 0.99 |
| cg23831517 | 8 | 34182528 | OpenSea | *-* | - | 0.04 | 0.00008 | 0.99 |
| cg04683824 | 16 | 34293670 | N_Shore | *-* | - | 0.04 | 0.00008 | 0.99 |
| cg03975690 | 7 | 87848292 | N_Shore | *SRI* | Body | -0.01 | 0.00008 | 0.99 |
| cg19202384 | 17 | 79894511 | N_Shore | *PYCR1* | Body | -0.02 | 0.00008 | 0.99 |
| cg25395505 | 7 | 97877765 | N_Shelf | *TECPR1* | 5'UTR | 0.02 | 0.00008 | 0.99 |

Abbreviations: Chr, chromosome;  FC, fold change; q-value, False discovery rate (FDR)-adjusted p-value using the Benjamini-Hochberg method.

Notes: The CpG sites were ranked in ascending order based on their unadjusted p-value (*); ^1^The models were adjusted for age, BMI and estimated cell proportions.

**Table S3. Top 20 CpG sites showing methylation changes associated with PFNA concentrations*.**

| **CpG** | **Chr** | **Position** | **CpG location** | **Genes** | **Gene region** | **logFC** | **p-value** | **q-value** |
| --- | --- | --- | --- | --- | --- | --- | --- | --- |
| cg15096505 | 1 | 206943566 | OpenSea | *IL10* | Body | -0.02 | 0.00001 | 0.99 |
| cg26825848 | 4 | 190566175 | N_Shore | *-* | - | -0.01 | 0.00002 | 0.99 |
| cg06390077 | 7 | 141385142 | OpenSea | *KIAA1147* | Body | -0.01 | 0.00003 | 0.99 |
| cg01710886 | 4 | 2819707 | Island | *SH3BP2* | TSS1500 | -0.04 | 0.00003 | 0.99 |
| cg23067082 | 12 | 7073179 | OpenSea | *MIR141* | TSS200 | 0.01 | 0.00003 | 0.99 |
| cg01824791 | 3 | 12875605 | OpenSea | *CAND2* | 3'UTR | -0.01 | 0.00004 | 0.99 |
| cg18151425 | 2 | 69093574 | OpenSea | *BMP10* | Body | 0.02 | 0.00004 | 0.99 |
| cg08136432 | 16 | 88902276 | Island | *GALNS* | Body | -0.20 | 0.00005 | 0.99 |
| cg02951765 | 16 | 65341412 | OpenSea | *LOC283867* | Body | 0.04 | 0.00005 | 0.99 |
| cg01448551 | 6 | 32798482 | OpenSea | *TAP2* | Body | -0.01 | 0.00005 | 0.99 |
| cg05641650 | 11 | 35693836 | OpenSea | *TRIM44* | Body | -0.01 | 0.00006 | 0.99 |
| cg20985635 | 5 | 132947278 | Island | *FSTL4* | 5'UTR | -0.01 | 0.00006 | 0.99 |
| cg19315508 | 1 | 149899669 | Island | *SF3B4* | 1stExon | 0.01 | 0.00006 | 0.99 |
| cg02445292 | 19 | 40417583 | N_Shelf | *FCGBP* | Body | -0.01 | 0.00007 | 0.99 |
| cg05014837 | 2 | 135059316 | OpenSea | *MGAT5* | Body | -0.01 | 0.00007 | 0.99 |
| cg14388195 | 17 | 1546961 | Island | *SCARF1* | Body | 0.01 | 0.00008 | 0.99 |
| cg13350173 | 19 | 38281183 | Island | *-* | - | -0.04 | 0.00008 | 0.99 |
| cg01614041 | 6 | 34123414 | S_Shore | *-* | - | -0.05 | 0.00008 | 0.99 |
| cg27455890 | 10 | 118976677 | Island | *-* | - | -0.04 | 0.00008 | 0.99 |
| cg20269046 | 7 | 149469722 | Island | *ZNF467* | 5'UTR | -0.17 | 0.00011 | 0.99 |

Abbreviations: Chr, chromosome;  FC, fold change; q-value, False discovery rate (FDR)-adjusted p-value using the Benjamini-Hochberg method.

Notes: The CpG sites were ranked in ascending order based on their unadjusted p-value (*); ^1^The models were adjusted for age, BMI and estimated cell proportions.

**Table S4. Top 20 CpG sites showing methylation changes associated with PFHxS concentrations*.**

| **CpG** | **Chr** | **Position** | **CpG location** | **Genes** | **Gene region** | **logFC** | **p-value** | **q-value** |
| --- | --- | --- | --- | --- | --- | --- | --- | --- |
| cg21879272 | 14 | 51135303 | Island | *SAV1* | TSS1500 | -0.05 | 0.00000 | 0.21 |
| cg19700328 | 14 | 106028568 | S_Shelf |  |  | -0.08 | 0.00000 | 0.44 |
| cg01458752 | 11 | 8008673 | N_Shore | *EIF3F* | TSS200 | -0.01 | 0.00000 | 0.44 |
| cg05228110 | 19 | 552948 | S_Shore |  |  | -0.02 | 0.00000 | 0.49 |
| cg10140583 | 8 | 143868110 | OpenSea | *LY6D* | TSS200 | -0.02 | 0.00003 | 0.99 |
| cg25435332 | 11 | 107328525 | Island | *CWF19L2* | 1stExon | -0.02 | 0.00003 | 0.99 |
| cg11791914 | 1 | 4772257 | Island | *AJAP1* | Body | -0.01 | 0.00003 | 0.99 |
| cg01035869 | 6 | 31242584 | S_Shelf |  |  | -0.02 | 0.00003 | 0.99 |
| cg00046623 | 8 | 52320263 | N_Shore | *PXDNL* | Body | 0.01 | 0.00004 | 0.99 |
| cg20166438 | 1 | 110091162 | Island | *GNAI3* | TSS200 | -0.003 | 0.00004 | 0.99 |
| cg10437839 | 11 | 64701910 | OpenSea | *PPP2R5B* | 3'UTR | 0.01 | 0.00005 | 0.99 |
| cg05318026 | 18 | 45936057 | Island |  |  | -0.01 | 0.00005 | 0.99 |
| cg12529083 | 12 | 13197360 | Island | *KIAA1467* | 1stExon | -0.005 | 0.00006 | 0.99 |
| cg25424279 | 11 | 65683543 | N_Shore |  |  | -0.01 | 0.00006 | 0.99 |
| cg03975690 | 7 | 87848292 | N_Shore | *SRI* | Body;Body | -0.01 | 0.00006 | 0.99 |
| cg23173910 | 2 | 74120274 | OpenSea | *ACTG2* | 5'UTR | 0.02 | 0.00006 | 0.99 |
| cg09002231 | 13 | 46036179 | N_Shelf |  |  | -0.01 | 0.00006 | 0.99 |
| cg11403706 | 22 | 49042600 | Island | *FAM19A5* | Body;Body | -0.01 | 0.00006 | 0.99 |
| cg12602945 | 19 | 49541529 | OpenSea | *CGB1* | TSS1500 | -0.03 | 0.00007 | 0.99 |
| cg16553052 | 7 | 2349605 | OpenSea | *SNX8* | Body | -0.01 | 0.00007 | 0.99 |

Abbreviations: Chr, chromosome;  FC, fold change; q-value, False discovery rate (FDR)-adjusted p-value using the Benjamini-Hochberg method.

Notes: The CpG sites were ranked in ascending order based on their unadjusted p-value (*); ^1^The models were adjusted for age, BMI and estimated cell proportions.

**Table S5. Top 20 CpG sites showing methylation changes associated with PFHpS concentrations*.**

| **CpG** | **Chr** | **Position** | **CpG location** | **Genes** | **Gene region** | **logFC** | **p-value** | **q-value** |
| --- | --- | --- | --- | --- | --- | --- | --- | --- |
| cg15932610 | 12 | 108820116 | OpenSea | - | - | 0.01 | <0.00001 | 0.40 |
| cg14883605 | 14 | 57021828 | OpenSea | - | - | 0.02 | <0.00001 | 0.40 |
| cg15063150 | 2 | 239133353 | OpenSea | - | - | 0.01 | <0.00001 | 0.40 |
| cg06311795 | 12 | 130818957 | N_Shelf | - | - | -0.01 | <0.00001 | 0.45 |
| cg04303139 | 7 | 94286753 | S_Shore | *SGCE;PEG10* | TSS1500;5'UTR | -0.01 | <0.00001 | 0.45 |
| cg22663489 | 11 | 64107720 | N_Shelf | *CCDC88B* | 1stExon | -0.02 | 0.00001 | 0.47 |
| cg01458752 | 11 | 8008673 | N_Shore | *EIF3F* | TSS200 | -0.01 | 0.00001 | 0.47 |
| cg13396436 | 6 | 32041568 | OpenSea | *TNXB* | Body | -0.01 | 0.00001 | 0.47 |
| cg20956314 | 3 | 38387299 | N_Shore | *XYLB* | TSS1500 | 0.01 | 0.00001 | 0.47 |
| cg26503018 | 7 | 94286243 | Island | *SGCE;PEG10* | TSS1500;5'UTR | -0.01 | 0.00001 | 0.47 |
| cg05819416 | 1 | 214140498 | OpenSea |  |  | 0.01 | 0.00001 | 0.47 |
| cg20055841 | 14 | 52780919 | Island | *PTGER2* | TSS200 | -0.003 | 0.00002 | 0.47 |
| cg11819121 | 21 | 37664031 | N_Shelf | *DOPEY2* | Body | 0.01 | 0.00002 | 0.47 |
| cg18086592 | 12 | 13153464 | Island | *HTR7P;HEBP1* | Body;TSS1500 | -0.01 | 0.00002 | 0.47 |
| cg22626548 | 5 | 2253129 | OpenSea |  |  | 0.01 | 0.00002 | 0.47 |
| cg08270005 | 8 | 96179452 | OpenSea |  |  | 0.01 | 0.00002 | 0.47 |
| cg21160562 | 4 | 183308763 | OpenSea | *ODZ3* | Body | -0.01 | 0.00002 | 0.47 |
| cg21039597 | 4 | 1005035 | Island | *FGFRL1* | TSS1500 | 0.004 | 0.00002 | 0.47 |
| cg01683270 | 4 | 8136996 | OpenSea | *ABLIM2* | Body | 0.01 | 0.00002 | 0.47 |
| cg27366072 | 12 | 12222913 | OpenSea | *BCL2L14* | TSS1500 | 0.01 | 0.00002 | 0.47 |

Abbreviations: Chr, chromosome;  FC, fold change; q-value, False discovery rate (FDR)-adjusted p-value using the Benjamini-Hochberg method.

Notes: The CpG sites were ranked in ascending order based on their unadjusted p-value (*); ^1^The models were adjusted for age, BMI and estimated cell proportions.

**Table S6. Top 20 CpG sites showing methylation changes associated with PFOS concentrations*.**

| **CpG** | **Chr** | **Position** | **CpG location** | **Genes** | **Gene region** | **logFC** | **p-value** | **q-value** |
| --- | --- | --- | --- | --- | --- | --- | --- | --- |
| cg23121615 | 8 | 144176359 | OpenSea |  |  | -0.04 | <0.000001 | 0.60 |
| cg23773680 | X | 8433466 | OpenSea | *VCX3B* | 5'UTR | -0.01 | <0.000001 | 0.60 |
| cg02187988 | 7 | 138677596 | OpenSea |  |  | 0.01 | 0.00001 | 0.72 |
| cg06605704 | 16 | 4551147 | OpenSea | *HMOX2* | 5'UTR | 0.01 | 0.00001 | 0.72 |
| cg26027442 | 14 | 104580345 | N_Shelf |  |  | -0.02 | 0.00001 | 0.72 |
| cg16498741 | 8 | 41144520 | OpenSea | *SFRP1* | Body | 0.01 | 0.00001 | 0.72 |
| cg27127645 | 14 | 55220956 | OpenSea | *SAMD4A* | TSS1500 | 0.01 | 0.00001 | 0.72 |
| cg09938674 | 8 | 1534838 | OpenSea | *DLGAP2* | Body | -0.01 | 0.00002 | 0.72 |
| cg18686270 | 3 | 146258875 | N_Shelf | *PLSCR1* | 5'UTR | 0.03 | 0.00002 | 0.72 |
| cg23796578 | 17 | 9694472 | OpenSea | *DHRS7C* | 1stExon | -0.02 | 0.00002 | 0.72 |
| cg21446955 | 4 | 86851425 | OpenSea | *ARHGAP24* | 5'UTR | -0.01 | 0.00002 | 0.72 |
| cg21452281 | 4 | 189203965 | Island |  |  | -0.02 | 0.00002 | 0.72 |
| cg19602139 | 3 | 49690254 | Island | *BSN* | Body | 0.01 | 0.00002 | 0.72 |
| cg03094519 | 17 | 37761662 | N_Shore | *NEUROD2* | 3'UTR | 0.01 | 0.00002 | 0.72 |
| cg04125962 | 16 | 27476250 | OpenSea | *GTF3C1* | Body | -0.02 | 0.00003 | 0.72 |
| cg01008405 | 5 | 139039026 | N_Shore | *CXXC5* | 5'UTR | -0.02 | 0.00003 | 0.72 |
| cg08428266 | 7 | 55238412 | OpenSea | *EGFR* | 3'UTR | 0.02 | 0.00003 | 0.72 |
| cg10331038 | 1 | 175376028 | OpenSea | *TNR* | 5'UTR | 0.02 | 0.00004 | 0.72 |
| cg05173758 | 13 | 27335096 | Island | *GPR12* | TSS200 | -0.01 | 0.00004 | 0.72 |
| cg08610886 | X | 13397207 | Island |  |  | -0.02 | 0.00005 | 0.72 |

Abbreviations: Chr, chromosome;  FC, fold change; q-value, False discovery rate (FDR)-adjusted p-value using the Benjamini-Hochberg method.

Notes: The CpG sites were ranked in ascending order based on their unadjusted p-value (*); ^1^The models were adjusted for age, BMI and estimated cell proportions.

**Table S7. Top 20 CpG sites showing methylation changes associated with br-PFOS concentrations*.**

| **CpG** | **Chr** | **Position** | **CpG location** | **Genes** | **Gene region** | **logFC** | **p-value** | **q-value** |
| --- | --- | --- | --- | --- | --- | --- | --- | --- |
| cg16498741 | 8 | 41144520 | OpenSea | *SFRP1* | Body | 0.01 | <0.000001 | 0.99 |
| cg17945447 | 1 | 42384002 | Island | *HIVEP3* | 5'UTR | -0.03 | 0.00001 | 0.99 |
| cg05173758 | 13 | 27335096 | Island | *GPR12* | TSS200 | -0.01 | 0.00001 | 0.99 |
| cg07601058 | 1 | 1597720 | N_Shelf | *LOC728661;CDK11B* | Body;Body | -0.01 | 0.00001 | 0.99 |
| cg23796578 | 17 | 9694472 | OpenSea | *DHRS7C* | 1stExon | -0.02 | 0.00002 | 0.99 |
| cg01008405 | 5 | 139039026 | N_Shore | *CXXC5* | 5'UTR | -0.01 | 0.00002 | 0.99 |
| cg24435669 | 1 | 15546149 | OpenSea | *TMEM51* | Body | -0.01 | 0.00002 | 0.99 |
| cg21152269 | 14 | 55519107 | S_Shore | *MAPK1IP1L* | 5'UTR | -0.01 | 0.00003 | 0.99 |
| cg02187988 | 7 | 138677596 | OpenSea |  |  | 0.01 | 0.00003 | 0.99 |
| cg00489219 | 3 | 40566141 | Island | *ZNF621* | TSS1500 | -0.01 | 0.00004 | 0.99 |
| cg23773680 | X | 8433466 | OpenSea | *VCX3B* | 5'UTR | -0.01 | 0.00005 | 0.99 |
| cg01828650 | 7 | 112430614 | S_Shore | *TMEM168* | TSS200 | -0.005 | 0.00005 | 0.99 |
| cg01185921 | 10 | 116444463 | OpenSea | *ABLIM1* | TSS200 | -0.02 | 0.00005 | 0.99 |
| cg11518408 | 2 | 242760773 | Island |  |  | -0.02 | 0.00006 | 0.99 |
| cg15635600 | 21 | 45681834 | S_Shelf | *DNMT3L* | 5'UTR | -0.03 | 0.00006 | 0.99 |
| cg02189800 | 16 | 88597301 | N_Shore | *ZFPM1* | Body | 0.03 | 0.00006 | 0.99 |
| cg21011403 | 2 | 43302362 | OpenSea |  |  | 0.01 | 0.00007 | 0.99 |
| cg00405939 | 19 | 4045108 | Island |  |  | 0.01 | 0.00007 | 0.99 |
| cg27127645 | 14 | 55220956 | OpenSea | *SAMD4A* | TSS1500 | 0.01 | 0.00007 | 0.99 |
| cg07811074 | 2 | 98350847 | N_Shore | *ZAP70* | Body | -0.01 | 0.00007 | 0.99 |

Abbreviations: Chr, chromosome;  FC, fold change; q-value, False discovery rate (FDR)-adjusted p-value using the Benjamini-Hochberg method.

Notes: The CpG sites were ranked in ascending order based on their unadjusted p-value (*); ^1^The models were adjusted for age, BMI and estimated cell proportions.

**Table S8. Sensitivity analyses for evaluating the impact of smoking status on the associations between PFAS exposure and DNA methylation (n = 263).**

|  | **Model 1^#^**  **(n = 269)** | | | **Model 2 (Sensitivity)^*^**  **(n = 263)** | | |
| --- | --- | --- | --- | --- | --- | --- |
| **CpG** | **logFC** | **p-value** | **q-value** | **logFC** | **p-value** | **q-value** |
| cg00149684 | 0.01 | 0.00000000004 | 0.00002 | 0.01 | 0.0000000001 | 0.00003 |
| cg26047920 | -0.04 | 0.0000000001 | 0.00003 | -0.04 | 0.0000000001 | 0.00003 |
| cg13623384 | 0.02 | 0.000000009 | 0.0015 | 0.02 | 0.000000008 | 0.001 |
| cg08154280 | -0.01 | 0.00000002 | 0.002 | -0.01 | 0.0000001 | 0.01 |
| cg23834427 | 0.03 | 0.00000002 | 0.002 | 0.03 | 0.00000002 | 0.002 |
| cg15137396 | 0.02 | 0.00000009 | 0.007 | 0.02 | 0. 0.0000003 | 0.02 |
| cg23232361 | 0.01 | 0.0000002 | 0.01 | 0.01 | 0.0000007 | 0.03 |
| cg10296914 | 0.03 | 0.0000002 | 0.01 | 0.03 | 0.0000006 | 0.03 |
| cg18142228 | 0.02 | 0.0000002 | 0.01 | 0.02 | 0.0000001 | 0.01 |
| cg02790122 | 0.01 | 0.0000002 | 0.01 | 0.01 | 0.0000004 | 0.02 |
| cg12165223 | 0.03 | 0.0000003 | 0.01 | 0.03 | 0.000002 | 0.08 |
| cg07885191 | 0.02 | 0.000001 | 0.04 | 0.01 | 0.000007 | 0.14 |
| cg15499402 | -0.01 | 0.000001 | 0.04 | -0.01 | 0.000003 | 0.09 |
| cg23952828 | 0.01 | 0.000001 | 0.04 | 0.01 | 0.000004 | 0.10 |
| cg22254103 | 0.02 | 0.000001 | 0.04 | 0.02 | 0.000002 | 0.06 |
| cg25050332 | 0.03 | 0.000001 | 0.04 | 0.03 | 0.000003 | 0.10 |
| cg02941923 | 0.01 | 0.000001 | 0.04 | 0.01 | 0.000003 | 0.10 |
| cg15511516 | 0.02 | 0.000001 | 0.04 | 0.02 | 0.00003 | 0.30 |
| cg15693793 | -0.03 | 0.000002 | 0.04 | -0.03 | 0.000004 | 0.10 |
| cg02344868 | 0.02 | 0.000002 | 0.04 | 0.02 | 0.000004 | 0.11 |
| cg14468055 | 0.01 | 0.000002 | 0.045 | 0.01 | 0.00003 | 0.30 |

Abbreviations: FC, fold change; q-value, False discovery rate (FDR)-adjusted p-value using the Benjamini-Hochberg method.

Notes: **^#^** Model 1 included age, BMI, and estimated cell proportions as covariates; ^*^ Model 2 further included smoking status as covariate.

**Table S9. Top 10 enriched GO terms from the nominally significant (p-value < 0.001) and non-FDR-corrected CpG sites associated with PFAS exposure**.

| **GO ID** | **GO Term** | **Total number of genes in Term** | **Number of genes with methylation changes** | **p-value** | **q-value** | **Genes** |
| --- | --- | --- | --- | --- | --- | --- |
| BP |  |  |  |  |  |  |
| GO:2000138 | positive regulation of cell proliferation involved in heart morphogenesis | 6 | 5 | 0.0002 | 1 | *BMP10, RBPJ, SMAD4, PIM1, SOX9* |
| GO:2000136 | regulation of cell proliferation involved in heart morphogenesis | 18 | 9 | 0.0003 | 1 | *BMP10, RBPJ, ISL1, SMAD4, NOTCH1, PIM1, SOX9, TBX5, HAND2* |
| GO:2001222 | regulation of neuron migration | 41 | 15 | 0.0007 | 1 | *NRG3, UNC5D, CTNNA2,*  *DAB2IP, PHACTR1, ZNF609, FLNA, FLRT2, MDK, ULK4, SEMA6A, STAT3, CAMK2B, KIF20, RAPGEF2* |
| GO:0061323 | cell proliferation involved in heart morphogenesis | 19 | 9 | 0.0007 | 1 | *BMP10, RBPJ, ISL1, SMAD4, NOTCH1, PIM1, SOX9, TBX5, HAND2* |
| GO:2000617 | positive regulation of histone H3-K9 acetylation | 8 | 5 | 0.001 | 1 | *SMAD4, NAP1L2, PIH1D, PIWIL2, BRCA1* |
| GO:0070424 | regulation of nucleotide-binding oligomerization domain containing signaling pathway | 13 | 6 | 0.001 | 1 | *SLC15A4, BIRC3, HSPA1B, XIAP, SLC15A2, TLR4* |
| GO:2001224 | positive regulation of neuron migration | 15 | 7 | 0.003 | 1 | *DAB2IP, ZNF609, FLNA, MDK, SEMA6A, KIF20B, RAPGEF2* |
| GO:0035019 | somatic stem cell population maintenance | 42 | 14 | 0.004 | 1 | *YAP1, CDX2, TAF6L, SIX2, ZHX2, RBPJ, LRP5, SFRP1, SOX9, VPS72, NANOG, LBH, CUL4A, LDB2* |
| GO:0002385 | mucosal immune response | 32 | 7 | 0.004 | 1 | *C17orf99, IFNLR1, DEFA4, IFNL2, PIGR, RPL39, RAB17, H2BC12* |
| GO:2000615 | regulation of histone H3-K9 acetylation | 11 | 5 | 0.005 | 1 | *SMAD4, NAP1L2, PIH1D1, PIWIL2, BRCA1* |
| MF |  |  |  |  |  |  |
| GO:0003712 | transcription coregulator activity | 478 | 79 | 0.007 | 1 | *CITED2, YAP1, RBM14, DDX17, DRAP1, TAF6L, RBPMS, TRIM31, CTBP1, CTBP2, HDGFL1, ZXDB, DAXX, ZFPM1, WBP2NL, ASXL1, DNMT3A, DNMT3B, DTX1, JMJD1C, SNW1, PHF8, CAMTA1, MED13L, ZFPM2, KAT6B, TCERG1L, SS18L1, SND1, HCFC1, HMGB2, HMGA1, APEX1, IRF4, KMT5A, MECP2, NAB2, RERE, NFKBIB, CNOT2, NOTCH1, PAWR, BCOR, YY1AP1, MAML3, RCOR3, TRERF1, MTA3, PTPN14, RBBP8, CCND1, TRIM27, PRDM16, TFB2M, BRCA1, TACC1, TAF12, TFAP2A, TLE2, TLE3, TP53BP1, TSG101, BTG2, ZXDC, NCOA4, HMGA2, DPF3, ARID5B, MPND, CBX4, PIR, CBFA2T3, HYAL2, ACTN1, ACTN2, PER2, BUD31, LDB2, COPS2, LPXN, CDYL, RNF14, NCOR2, HDAC4* |
| GO:0004957 | prostaglandin E receptor activity | 5 | 3 | 0.008 | 1 | *HPGD, PTGER1, PTGER2* |
| GO:0035259 | glucocorticoid receptor binding | 14 | 6 | 0.009 | 1 | *GRIP1, NR4A2, STAT3, STAT5B, TACC1, NCOR2* |
| GO:0042975 | peroxisome proliferator activated receptor binding | 12 | 5 | 0.009 | 1 | *ASXL1, HMGA1, MDM2, NFATC4, TACC1* |
| GO:0003779 | actin binding | 436 | 78 | 0.01 | 1 | *MAEA, CAP2, SORBS1, GIPC1, IQGAP2, WASF3, FERMT2, FGD4, PACRG, MIB2, CTNNA2, PPP1R18, EEF2, EGFR, EPB41L2, DMTN, PHACTR1, ALDOA, DAAM1, PALLD, FLNA, MPRIP, FLNB, DAAM2, CORO1C, RUSC1, PANX1, FSCN2, RAI14, ABL2, FHOD1, TMOD2, WASH3P, KCNMA1, ABLIM1, MYBPC1, MYH9, MYL2, MYO9B, MYO10, PAWR, LIMA1, PLEC, SNTG1, SSH1, ANLN, INO80, EPS8L1, IMPACT, BAIAP2L1, FMN2, SPIRE1, CORO1B, PTK2, SHROOM3, KLHL1, AFAP1, MYO1G, SLC6A2, PHACTR4, DST, SVIL, TNNI2, UTRN, WIPF1, CORO7, MYH14, FHOD3, CAMK2B, CAPZA2, CAPZB, ANTXR1, ABLIM2, SCIN, ACTN1, ACTN2, IQGAP1, ACTN3, NEXN, MYOT* |
| GO:0008510 | sodium:bicarbonate symporter activity | 6 | 3 | 0.01 | 1 | *SLC4A10, SLC4A4, SLC4A7* |
| GO:0140078 | class I DNA-(apurinic or apyrimidinic site) endonuclease activity | 7 | 3 | 0.02 | 1 | *NEIL2, NTHL1, NEIL3* |
| GO:0003906 | DNA-(apurinic or apyrimidinic site) endonuclease activity | 12 | 4 | 0.02 | 1 | *NEIL2, APEX1, NTHL1, NEIL3, HMGA2* |
| GO:0030145 | manganese ion binding | 63 | 13 | 0.02 | 1 | *NUDT16, XXYLT1, ATP13A2, GALNT2, ABL2, GLUL, GYG1, MGAT5, PIM1, FAM20C, XYLT2, MPPE1, XPNPEP1* |
| GO:0019787 | ubiquitin-like protein transferase activity | 445 | 65 | 0.02 | 1 | *RNF41, MAEA, RBCK1, RFPL2, WWP1, UBE2C, TRIM31, MARCHF3, TRIM71, UBE2QL1, TRIM50, MIB2, RNF168, DTX1, TRIML2, LNX2, HECW1, FBXW11, MGRN1, TRIM2, NEDD4L, LTN1, FBXO24, FBXO3, FBXL22, RNF175, RNF180, UHRF1, BIRC3, XIAP, NCCRP1, MDM2, TRIM72, RNF165, NOSIP, TRIM17, ANKRD39, UFC1, PEX12, MARCHF5, TRIM44, BCOR, ATG16L1, RNF220, TRIM36, UBE2Q1, PELI1, RNF150, RNF213, TRIM27, BRCA1, PPP1R11, TRAF3, UBE2H, UBE2I, MUL1, RNF39, CUL5, ZFP91, KCTD10, CUL4A, TRIM51, CBX4, HERC2, UBE3B, UBE2M, UBE2L6, RNF14, HDAC4, RNF144A* |
| CC |  |  |  |  |  |  |
| GO:0032045 | guanyl-nucleotide exchange factor complex | 17 | 7 | 0.001 | 1 | *LAMTOR5, DOCK1, C9orf72, RASGRP3, KNDC1, EIF2B3, ELMO1* |
| GO:1902773 | GTPase activator complex | 18 | 7 | 0.002 | 1 | *LAMTOR5, DOCK1, C9orf72, RASGRP3, KNDC1, EIF2B3, ELMO1* |
| GO:0070938 | contractile ring | 10 | 5 | 0.007 | 1 | *MAEA, MYH9, ANLN, UTRN, KIF20B* |
| GO:0097433 | dense body | 6 | 4 | 0.008 | 1 | *SND1, PIWIL2, SYBU, ACTG1* |
| GO:0019814 | immunoglobulin complex | 7 | 3 | 0.009 | 1 | *PIGR, LIME1, CD79B* |
| GO:0031315 | extrinsic component of mitochondrial outer membrane | 2 | 2 | 0.01 | 1 | *SARM1, SOX10* |
| GO:0005769 | early endosome | 390 | 63 | 0.01 | 1 | *ATP9A, FLOT1, STX6, MARCHF3, CLCN4, CLCN5, SLC15A4, MIB2, PHETA2, CLVS1, SNX31, EGFR, EPHA4, LMTK2, MON2, ASTN2, ATP11A, MGRN1, RUSC1, LAMP5, PCSK9, CNTNAP2, ANK2, SNX8, SNX15, HLA-A, HLA-B, HLA-H, MR1, APBB2, APOB, WASH3P, KDR, KIFC1, SLC11A2, DERL2, CCDC93, CMTM6, APPL2, LAPTM4B, KIF16B, TMEM127, VAC14, MAP2K2, TMEM9B, GRIPAP1, ZFYVE28, RAB5C, TRIM27, VIPAS39, RAB17, SH3GL3, SLC5A1, TLR4, TSG101, PLEKHF2, RABEP2, RIN3, RND2, AOC3, SNX3, MVB12B, HAP1, DOP1B* |
| GO:0005826 | Actomyosin contractile ring | 4 | 3 | 0.02 | 1 | *MAEA, MYH9, ANLN* |
| GO:0098685 | Schaffer collateral - CA1 synapse | 78 | 18 | 0.02 | 1 | *CACNG3, BAIAP2, GIPC1, CPLX1, EPHA4, SYT11, GABBR1, ABR, GRM1, PLAT, PRKAR1B, PTPRD, PTPRS, STAT3, SYP, ACTG1, TNR, CAPZB, BSN* |
| GO:0031301 | integral component  of organelle membrane | 375 | 57 | 0.02 | 1 | *BCAP31, STX6, SLC15A4, FITM2, XXYLT1, SPPL2C, SYNE4, ABCD1, LEMD2, DOLK, P2RX2, ANKLE2, ATP13A2, GABRA2, SUN3, GALNT2, LRIT1, BSCL2, SLC46A3, COA3, HLA-A, HLA-B, HLA-DPA1, HLA-DPB1, HLA-DQA2, HLA-H, LFNG, NPC1, P2RX5, DERL2, ARMCX3, PIGT, ERGIC3, PEX12, A4GALT, PCSK4, PIGG, FAR2, SPIRE1, ERGIC1, PTPRN2, PTPRS, ELOVL5, RYR1, BNIP3, SYP, SYT1, SYT5, TAP2, TAPBP, SLC35A2, APOO, MUL1, DGAT2, STX10, GPAA1, MCU, SYNGR1, SMDT1, ITM2B* |

Abbreviations: GO, Gene Ontology; BP, Biological Process; MF, Molecular Function; CC, Cellular Component.

**Table S10. Top 20 genes with significant expression changes associated with methylation changes at specific CpG sites in response to PFAS exposure.**

| **PFAS** | **CpG associated** | **DNA methylation logFC** | **DNA methylation**  **p-value** | **DNA methylation**  **q-value** | **Annotated gene** | **Gene expression logFC** | **Gene expression p-value** | **Gene expression q-value** |
| --- | --- | --- | --- | --- | --- | --- | --- | --- |
| PFHpS | *cg15032638* | -0.005 | 0.00005 | 0.47 | *NCOA4* | -0.15 | 0.0008 | 0.1 |
| PFHpS | *cg15962031* | -0.008 | 0.0008 | 0.55 | *SMYD3* | 0.075 | 0.002 | 0.1 |
| PFHxS | *cg15520941* | 0.02 | 0.0008 | 0.99 | *C1orf124* | -0.09 | 0.003 | 0.4 |
| PFHpS | *cg19995112* | -0.006 | 0.0008 | 0.55 | *CSE1L* | -0.06 | 0.003 | 0.1 |
| PFOS | *cg02546490* | -0.01 | 0.0003 | 0.74 | *AATK* | -0.1 | 0.003 | 0.6 |
| PFOS | *cg18068798* | -0.01 | 0.0004 | 0.74 | *RND2* | -0.1 | 0.004 | 0.6 |
| PFOS | *cg26224354* | 0.03 | 0.0005 | 0.74 | *GPR146* | -0.3 | 0.005 | 0.6 |
| PFHpS | *cg07303187* | 0.01 | 0.0003 | 0.55 | *ZXDC* | -0.07 | 0.006 | 0.2 |
| PFHpS | *cg04462547* | -0.01 | 0.0004 | 0.55 | *PTPRJ* | -0.06 | 0.007 | 0.2 |
| PFHpS | *cg06512128* | -0.005 | 0.0004 | 0.55 | *RHOC* | 0.12 | 0.008 | 0.2 |
| PFHxS | *cg16025035* | -0.01 | 0.00008 | 0.99 | *TFDP1* | -0.16 | 0.009 | 0.5 |
| PFHpS | *cg24158452* | 0.004 | 0.0002 | 0.55 | *STAT5B* | -0.1 | 0.009 | 0.2 |
| PFOS | *cg04162497* | -0.02 | 0.0005 | 0.74 | *ZFYVE21* | -0.08 | 0.009 | 0.7 |
| PFHpS | *cg15148879* | -0.02 | 0.0003 | 0.55 | *FNBP4* | -0.08 | 0.01 | 0.2 |
| PFHpS | *cg05651393* | -0.008 | 0.001 | 0.56 | *NRD1* | -0.05 | 0.01 | 0.2 |
| PFHxS | *cg11732282* | -0.003 | 0.0005 | 0.99 | *ARL6IP6* | -0.12 | 0.01 | 0.6 |
| PFHpS | *cg24574546* | 0.008 | 0.0008 | 0.55 | *TMEM9B* | -0.08 | 0.01 | 0.2 |
| PFHpS | *cg11030124* | 0.01 | 0.0006 | 0.55 | *ASXL1* | -0.04 | 0.01 | 0.2 |
| PFHpS | *cg02276269* | -0.01 | 0.0002 | 0.55 | *UFC1* | -0.09 | 0.01 | 0.2 |
| PFHpS | *cg16346555* | 0.02 | 0.0005 | 0.55 | *ANTXR1* | -0.09 | 0.01 | 0.2 |

Abbreviations: FC, fold change; q-value, False discovery rate (FDR)-adjusted p-value using the Benjamini-Hochberg method.

Notes: The models included age, BMI, and estimated cell proportions as covariates. The grey shading highlights genes that showed changes in expression with a p-value < 0.01.

**Table S11. Estimates (β) for the association between PFAS concentrations (ng/mL) and estimated cell type proportions.**

| **PFAS** | **Monocytes** | **CD4 T-cells** | **CD8 T-cells** | **NK cells** | **B** | **Neutrophils** | **Eosinophils** |
| --- | --- | --- | --- | --- | --- | --- | --- |
| **PFOA** | -0.01 | 0.02 | 0 | 0 | **0.01^#^** | -0.02 | 0 |
| **PFNA** | 0 | 0.03 | -0.02 | 0 | 0 | -0.01 | 0 |
| **PFUnDA** | 0 | 0 | 0.01 | 0 | 0 | 0 | 0 |
| **PFHxS** | -0.01 | 0.01 | 0 | -0.01 | 0 | 0 | 0 |
| **PFHpS** | 0 | 0 | 0.01 | 0 | 0 | -0.01 | 0 |
| **PFOS** | 0 | 0.01 | 0 | 0.01 | 0 | -0.02 | 0 |
| **br-PFOS** | 0 | 0 | 0.01 | 0.01 | 0 | -0.02 | 0 |

Abbreviations: NK, natural killer.

Note: The models adjusted for age and BMI; ^#^ p-value < 0.05.


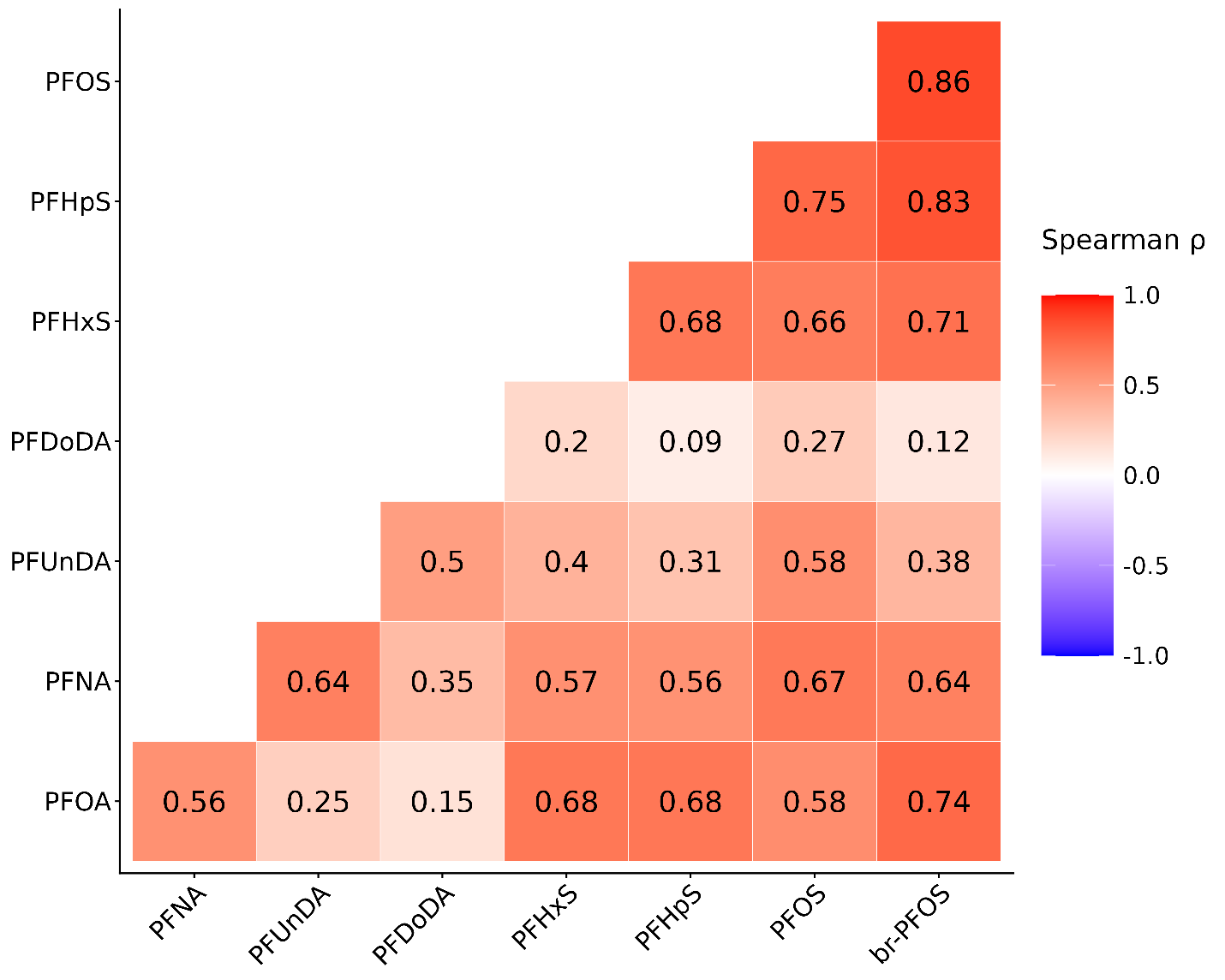


**Figure S1. Spearman rank correlation coefficients of plasma concentrations of PFAS compounds (ln-transformed).**


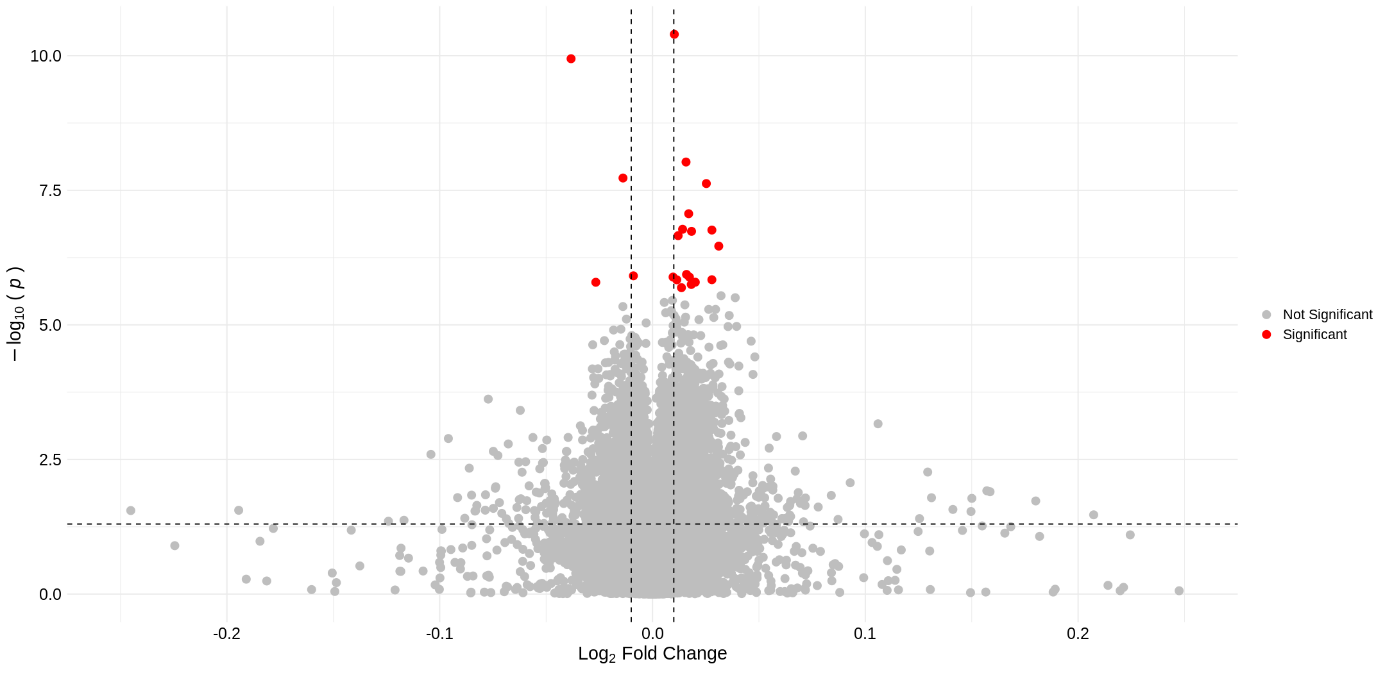


**Figure S2. Epigenome-wide identification of CpG methylation changes associated with PFUnDA exposure. Volcano plot showing the log_2_-fold change in methylation (*β* values) (x-axis) and the -log_10_ (p-value) from the epigenome-wide analysis (y-axis). Significant CpG sites with FDR < 0.05 are shown in red.**


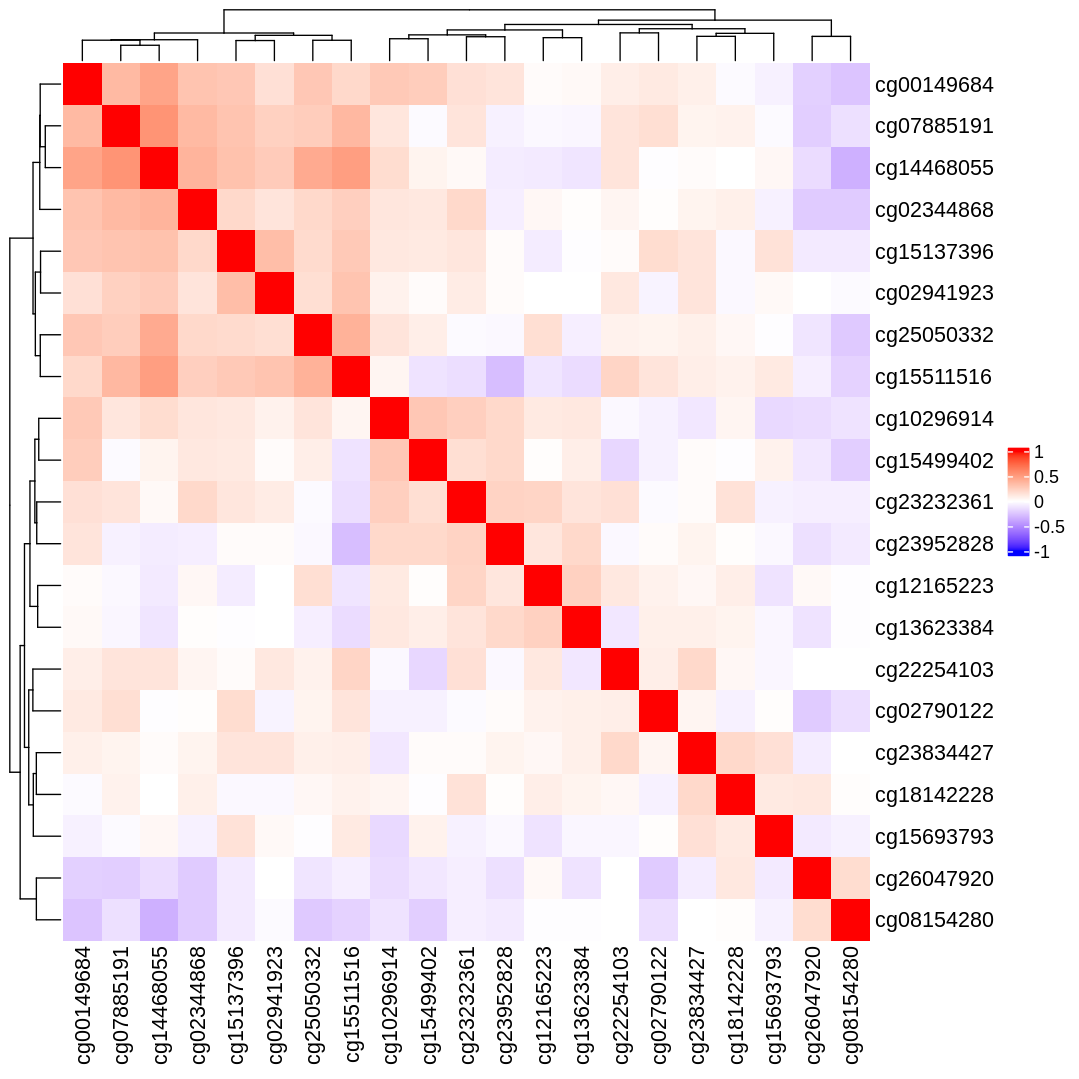


**Figure S3. Spearman correlation between CpG sites associated with PFUnDA.** Heatmap showing the correlation between significantly associated CpG sites. The strength of the correlation is represented by the color scale shown in the bar on the right.


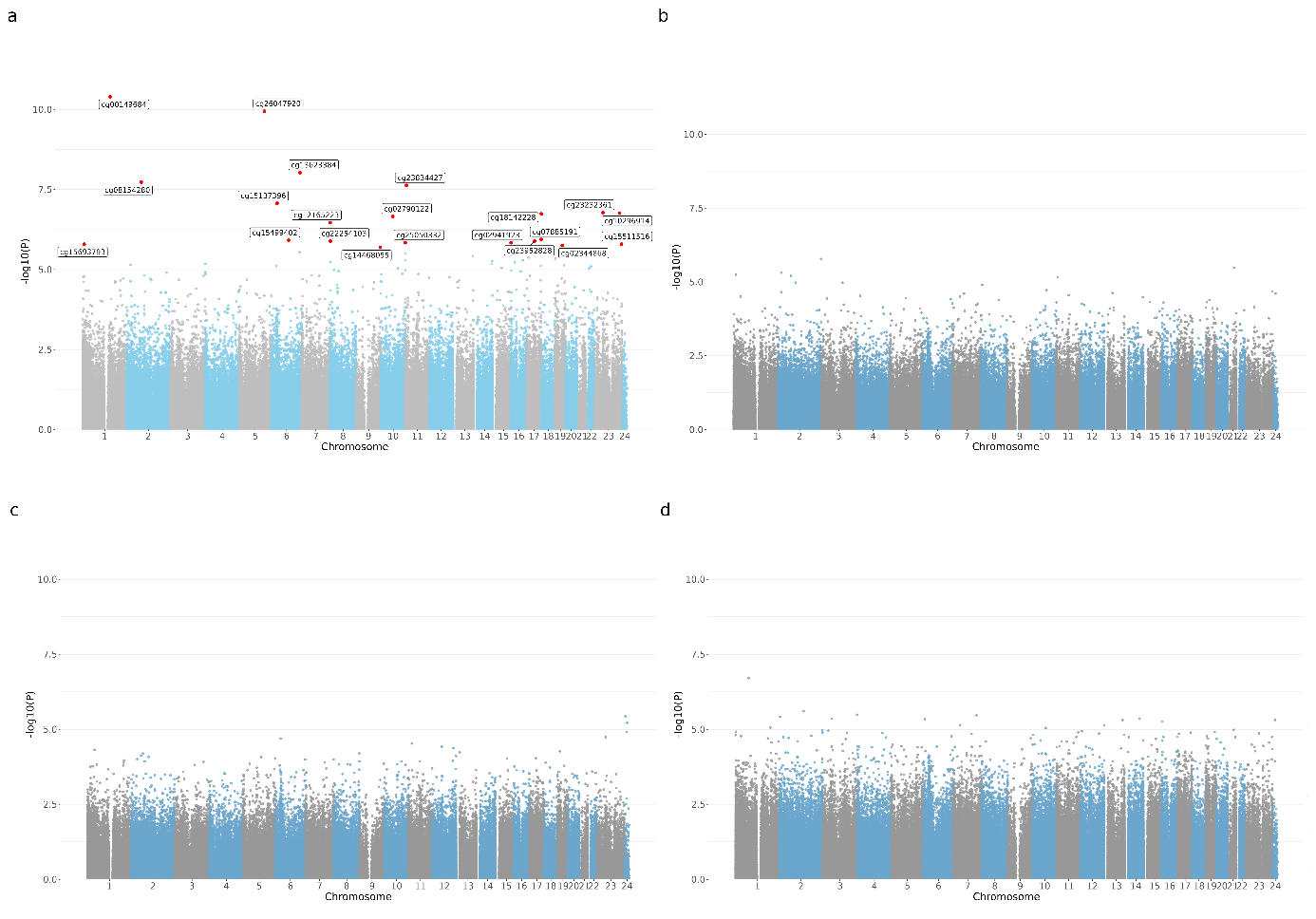


**Figure S4. Manhattan plots showing the associations between PFUnDA and DNA methylation at CpG sites**. The associations were tested using (a) PFAS concentrations as a continuous variable, and (b-d) tertile comparisons: tertile 3 vs. 1, tertile 3 vs. 2, and tertile 2 vs. 1. The x-axis represents genomic position across chromosomes, and the y-axis shows –log10(p-values) for each CpG site. No statistically significant associations were observed for PFHxS in either continuous or tertile analyses.


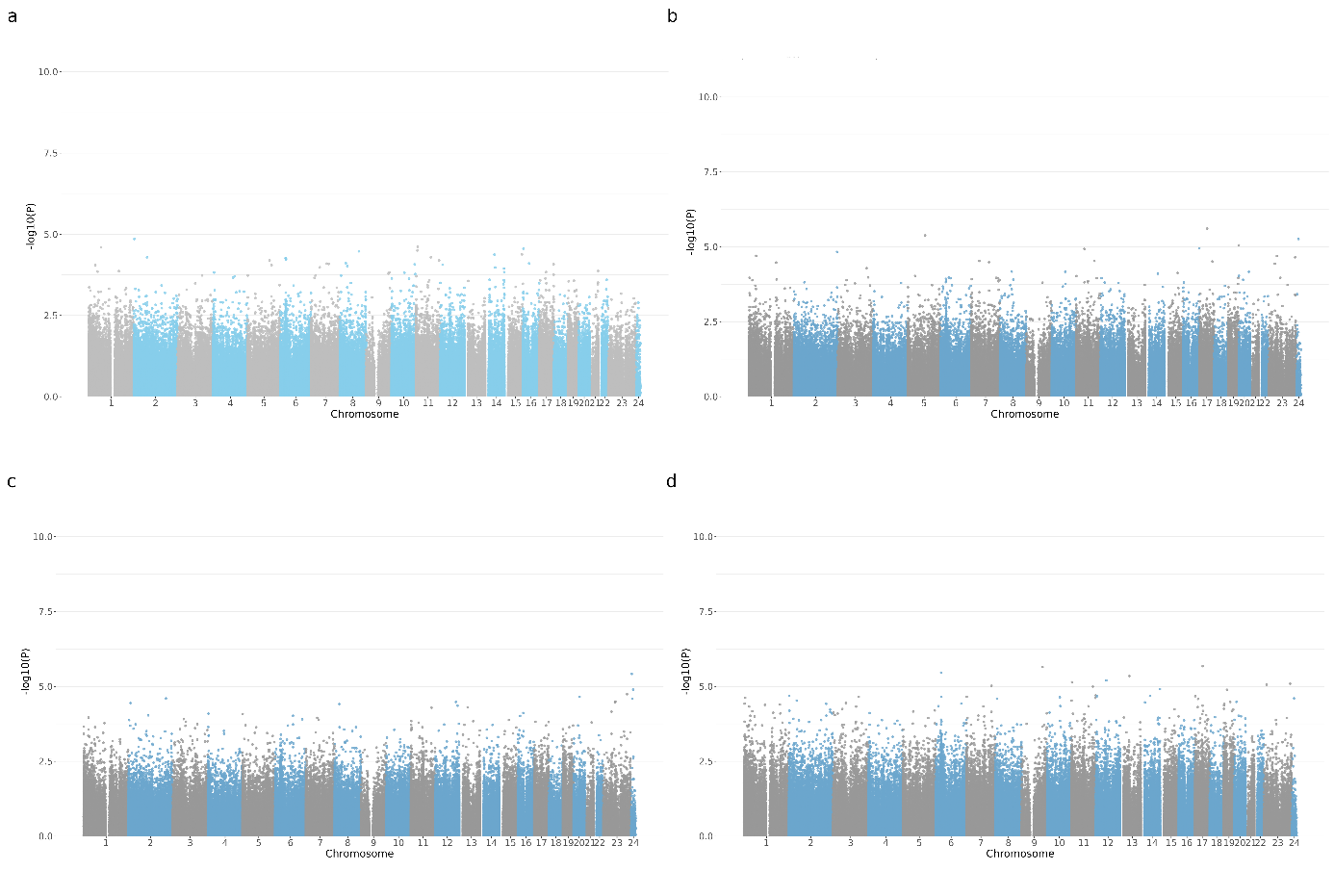


**Figure S5. Manhattan plots showing the associations between PFOA and DNA methylation at CpG sites.** The associations were tested using (a) PFAS concentrations as a continuous variable, and (b-d) tertile comparisons: tertile 3 vs. 1, tertile 3 vs. 2, and tertile 2 vs. 1. The x-axis represents genomic position across chromosomes, and the y-axis shows –log10(p-values) for each CpG site. No statistically significant associations were observed for PFOA in either continuous or tertile analyses.


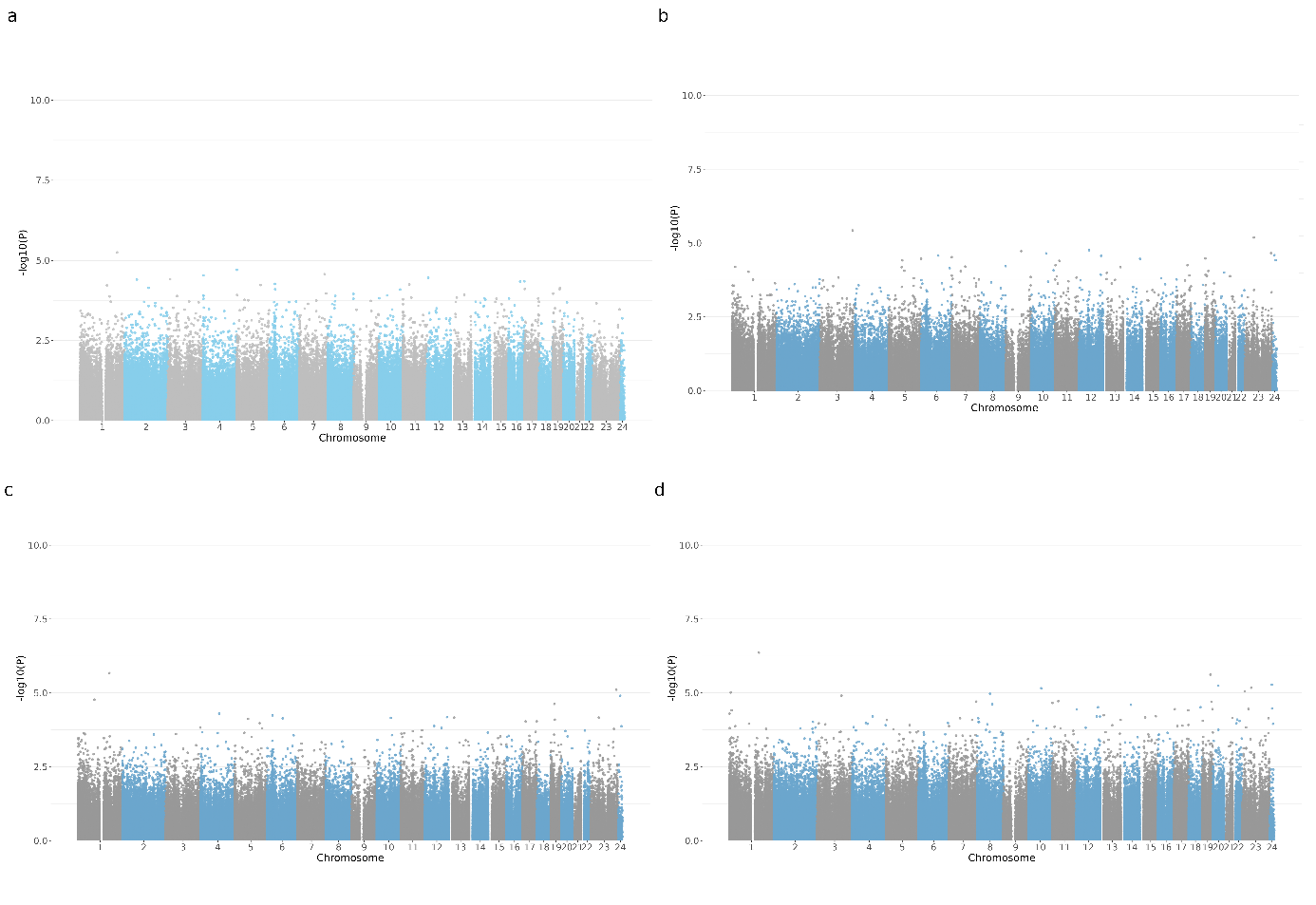


**Figure S6. Manhattan plots showing the associations between PFNA and DNA methylation at CpG sites.** The associations were tested using (a) PFAS concentrations as a continuous variable, and (b-d) tertile comparisons: tertile 3 vs. 1, tertile 3 vs. 2, and tertile 2 vs. 1. The x-axis represents genomic position across chromosomes, and the y-axis shows –log10(p-values) for each CpG site. No statistically significant associations were observed for PFNA in either continuous or tertile analyses.


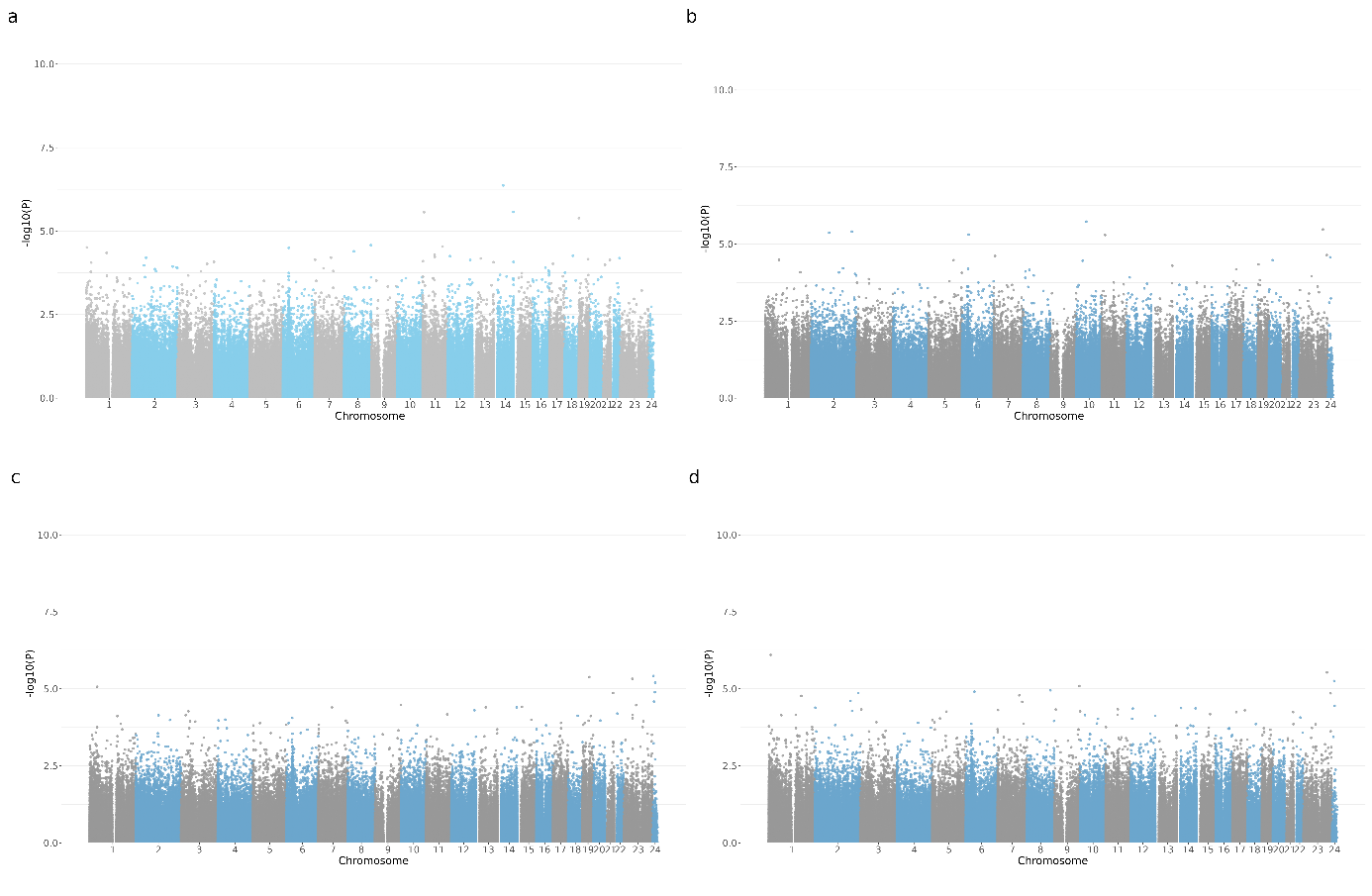


**Figure S7. Manhattan plots showing the associations between PFHxS and DNA methylation at CpG sites**. The associations were tested using (a) PFAS concentrations as a continuous variable, and (b-d) tertile comparisons: tertile 3 vs. 1, tertile 3 vs. 2, and tertile 2 vs. 1. The x-axis represents genomic position across chromosomes, and the y-axis shows –log10(p-values) for each CpG site. No statistically significant associations were observed for PFHxS in either continuous or tertile analyses.


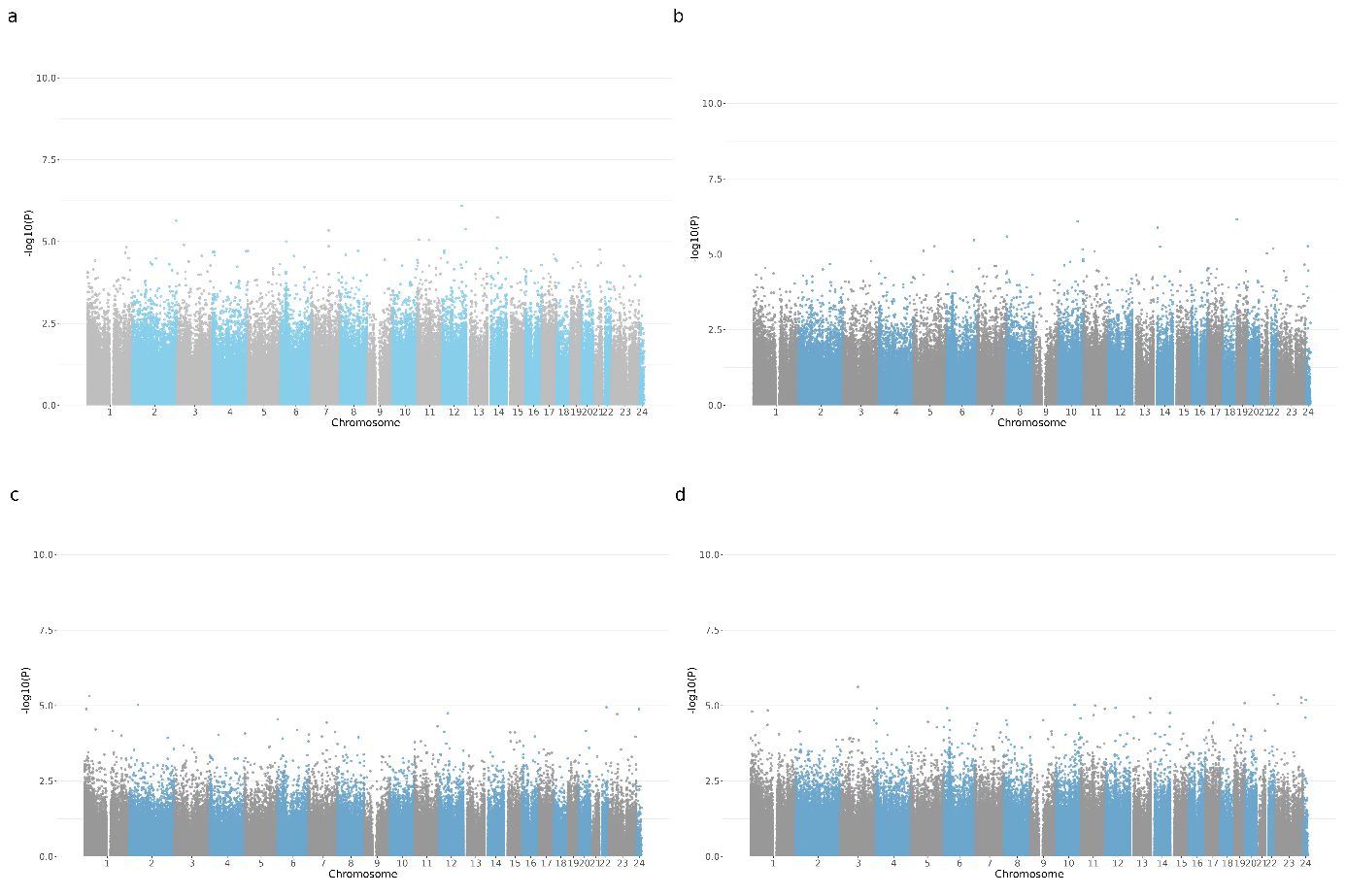


**Figure S8. Manhattan plots showing the associations between PFHpS and DNA methylation at CpG sites**. The associations were tested using (a) PFAS concentrations as a continuous variable, and (b-d) tertile comparisons: tertile 3 vs. 1, tertile 3 vs. 2, and tertile 2 vs. 1. The x-axis represents genomic position across chromosomes, and the y-axis shows –log10(p-values) for each CpG site. No statistically significant associations were observed for PFHpS in either continuous or tertile analyses.


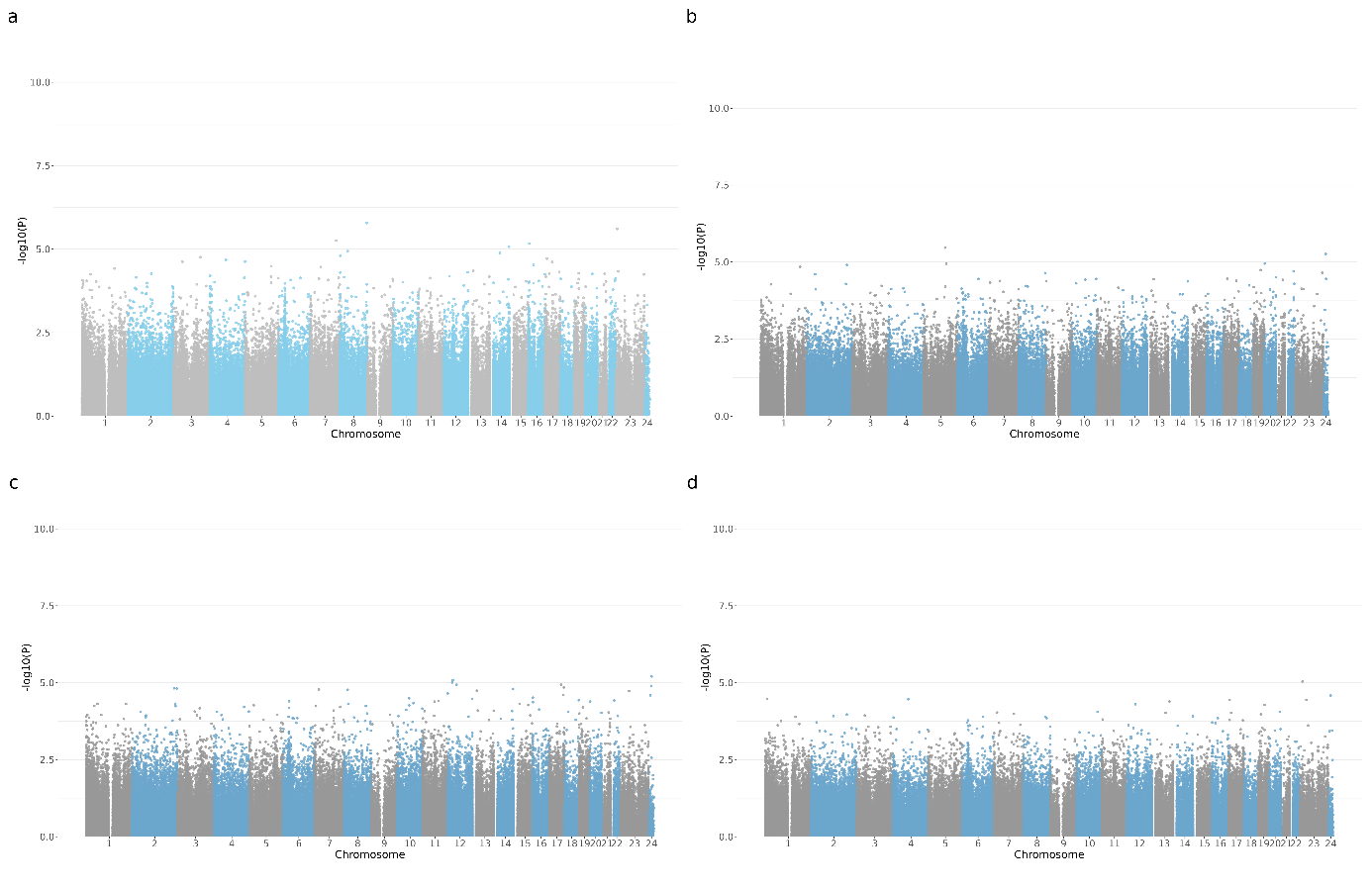


**Figure S9. Manhattan plots showing the associations between PFOS and DNA methylation at CpG sites**. The associations were tested using (a) PFAS concentrations as a continuous variable, and (b-d) tertile comparisons: tertile 3 vs. 1, tertile 3 vs. 2, and tertile 2 vs. 1. The x-axis represents genomic position across chromosomes, and the y-axis shows –log10(p-values) for each CpG site. No statistically significant associations were observed for PFOS in either continuous or tertile analyses.


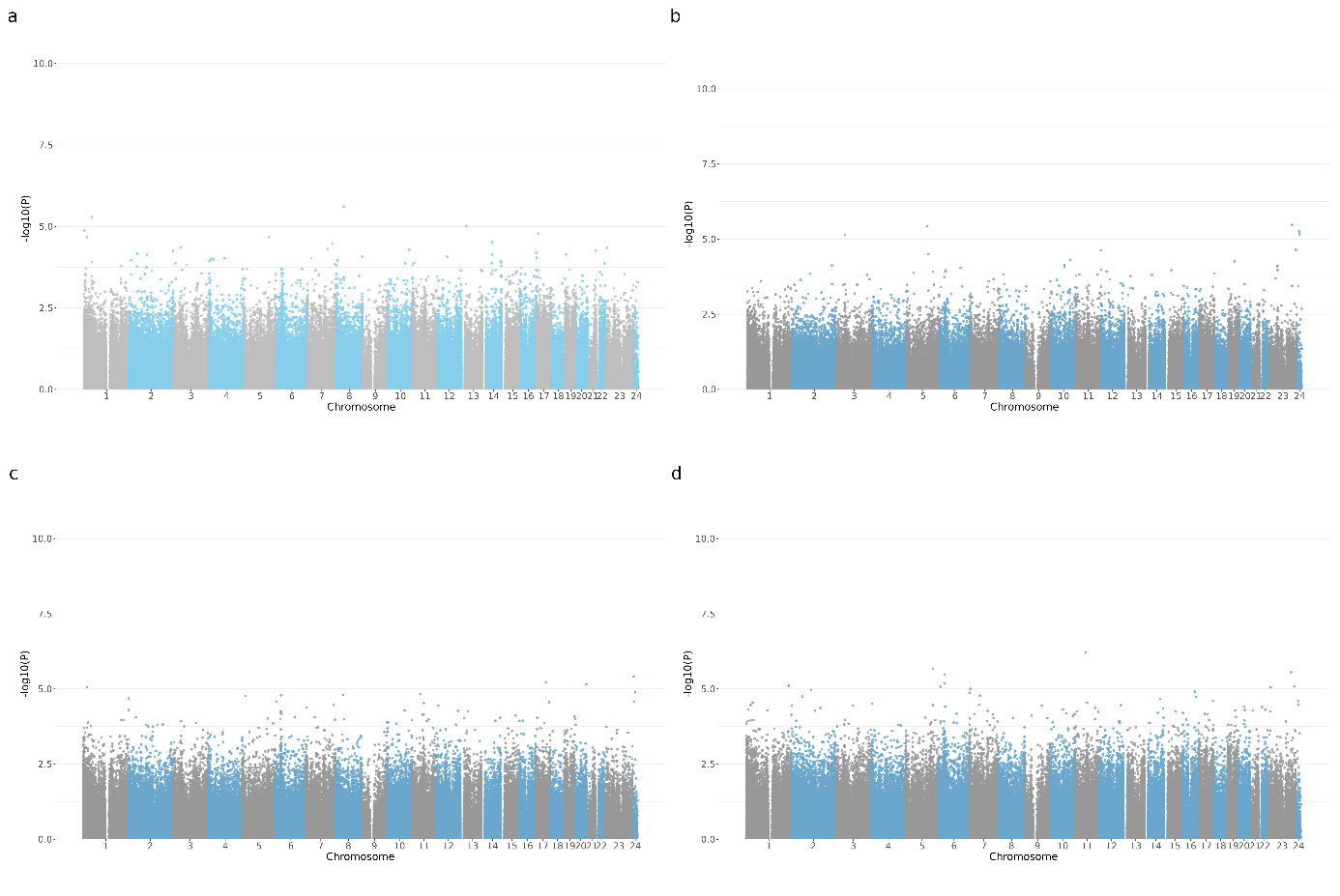


**Figure S10. Manhattan plots showing the associations between br-PFOS and DNA methylation at CpG sites**. The associations were tested using (a) PFAS concentrations as a continuous variable, and (b-d) tertile comparisons: tertile 3 vs. 1, tertile 3 vs. 2, and tertile 2 vs. 1. The x-axis represents genomic position across chromosomes, and the y-axis shows –log10(p-values) for each CpG site. No statistically significant associations were observed for br-PFOS in either continuous or tertile analyses.

**
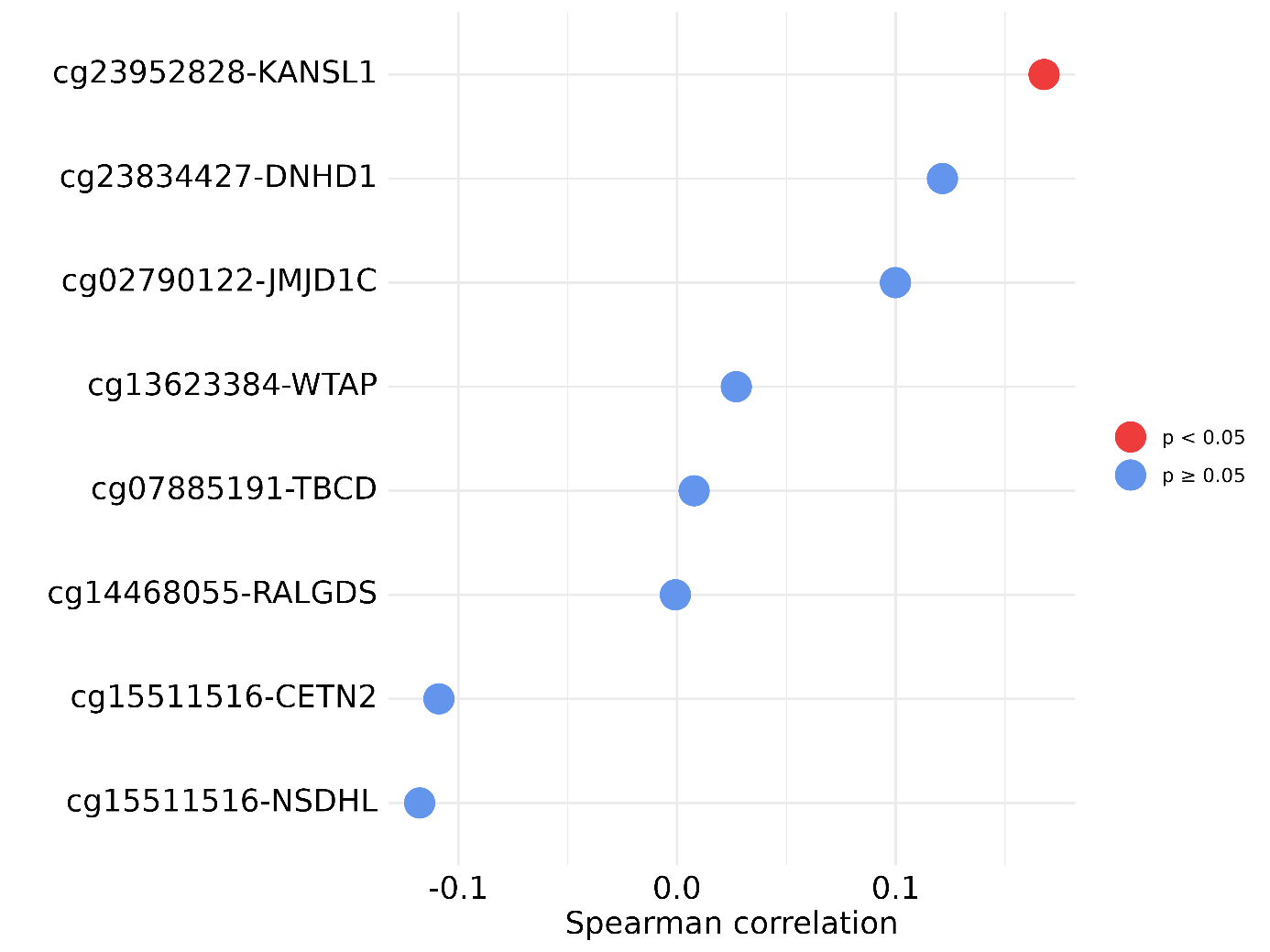
**

**Figure S11. Spearman correlations between CpG site–gene pairs.** Correlations were calculated between DNA methylation levels at CpG sites and expression levels of the corresponding genes.
